# Personalization of Conversational Agent-Patient Interaction Styles for Chronic Disease Management: Two Consecutive Cross-sectional Questionnaire Studies

**DOI:** 10.2196/26643

**Published:** 2021-05-26

**Authors:** Christoph Gross, Theresa Schachner, Andrea Hasl, Dario Kohlbrenner, Christian F Clarenbach, Forian V Wangenheim, Tobias Kowatsch

**Affiliations:** 1 Department of Management, Technology, and Economics ETH Zurich Zurich Switzerland; 2 Centre for Digital Health Interventions Department of Management, Technology, and Economics ETH Zurich Zurich Switzerland; 3 Department of Educational Sciences University of Potsdam Potsdam Germany; 4 International Max Planck Research School on the Life Course Berlin Germany; 5 Department of Pulmonology University Hospital Zurich Zurich Switzerland; 6 Faculty of Medicine University of Zurich Zurich Switzerland; 7 Centre for Digital Health Interventions Institute of Technology Management University of St. Gallen St. Gallen Switzerland; 8 Saw Swee Hock School of Public Health National University of Singapore Singapore Singapore

**Keywords:** conversational agents, chatbots, human-computer interaction, physician-patient interaction styles, deliberative interaction, paternalistic interaction, digital health, chronic conditions, disease management, COPD, chronic obstructive pulmonary disease

## Abstract

**Background:**

Conversational agents (CAs) for chronic disease management are receiving increasing attention in academia and the industry. However, long-term adherence to CAs is still a challenge and needs to be explored. Personalization of CAs has the potential to improve long-term adherence and, with it, user satisfaction, task efficiency, perceived benefits, and intended behavior change. Research on personalized CAs has already addressed different aspects, such as personalized recommendations and anthropomorphic cues. However, detailed information on interaction styles between patients and CAs in the role of medical health care professionals is scant. Such interaction styles play essential roles for patient satisfaction, treatment adherence, and outcome, as has been shown for physician-patient interactions. Currently, it is not clear (1) whether chronically ill patients prefer a CA with a paternalistic, informative, interpretive, or deliberative interaction style, and (2) which factors influence these preferences.

**Objective:**

We aimed to investigate the preferences of chronically ill patients for CA-delivered interaction styles.

**Methods:**

We conducted two studies. The first study included a paper-based approach and explored the preferences of chronic obstructive pulmonary disease (COPD) patients for paternalistic, informative, interpretive, and deliberative CA-delivered interaction styles. Based on these results, a second study assessed the effects of the paternalistic and deliberative interaction styles on the relationship quality between the CA and patients via hierarchical multiple linear regression analyses in an online experiment with COPD patients. Patients’ sociodemographic and disease-specific characteristics served as moderator variables.

**Results:**

Study 1 with 117 COPD patients revealed a preference for the deliberative (50/117) and informative (34/117) interaction styles across demographic characteristics. All patients who preferred the paternalistic style over the other interaction styles had more severe COPD (three patients, Global Initiative for Chronic Obstructive Lung Disease class 3 or 4). In Study 2 with 123 newly recruited COPD patients, younger participants and participants with a less recent COPD diagnosis scored higher on interaction-related outcomes when interacting with a CA that delivered the deliberative interaction style (interaction between age and CA type: *relationship quality: b*=−0.77, 95% CI −1.37 to −0.18; *intention to continue interaction: b*=−0.49, 95% CI −0.97 to −0.01; *working alliance attachment bond: b*=−0.65, 95% CI −1.26 to −0.04; *working alliance goal agreement: b*=−0.59, 95% CI −1.18 to −0.01; interaction between recency of COPD diagnosis and CA type: *working alliance goal agreement: b*=0.57, 95% CI 0.01 to 1.13).

**Conclusions:**

Our results indicate that age and a patient’s personal disease experience inform which CA interaction style the patient should be paired with to achieve increased interaction-related outcomes with the CA. These results allow the design of personalized health care CAs with the goal to increase long-term adherence to health-promoting behavior.

## Introduction

The occurrence of chronic diseases is on the rise owing to greater longevity of the population, increasing exposure to environmental pollution, and unhealthy lifestyles [1]. As chronic diseases are not curable, related care is directed toward improving the functional status, reducing distressing symptoms, extending life duration through secondary prevention, and improving health-related quality of life [2,3]. This requires comprehensive and personalized disease management based on active long-term collaboration between health care practitioners and chronically ill patients [1].

However, disease management is time-consuming and staff-intensive and is thus often not sufficiently provided [1]. Conversational agents (CAs) (ie, computer programs that imitate interactions with humans) have the potential to improve the status quo as they allow for cheaper and scalable patient support outside the clinical setting [4,5]. When deployed on a smartphone, CAs remain easily accessible and can accompany patients in their daily lives [6,7]. However, long-term adherence to interventions delivered by health care CAs and the effectiveness of interventions with regard to health-related outcomes remain challenging [8,9].

To increase adherence and user value with respect to satisfaction, task efficiency, or the likelihood of sustained outcomes, personalization of CAs is viewed as promising [10]. Despite numerous design considerations [9] for health care CAs, such as personalized recommendations [10] and anthropomorphic cues [11], it is still unclear which CA-delivered interaction styles chronically ill patients prefer and whether the preference has an impact on CA-related perceptions (eg, working alliance) and health outcomes (eg, change in health-promoting behaviors). Research has singled out the importance of the interaction style for treatment satisfaction, adherence, and subsequent outcome [12-14] in face-to-face encounters between physicians and patients and in distance therapy via the phone, internet, or other means [15]. As people apply social behavior and expectations to computers or other media in the presence of anthropomorphic cues (“computers are social actors” paradigm) [16], CA-delivered interaction styles are expected to be of high relevance.

This paper applies and investigates the following four interaction styles of health care CAs [17]: (1) paternalistic (the physician, as a guardian [17], decides alone about the most appropriate treatment based on the assumption of shared values); (2) informative (the physician, as an expert [17], neutrally provides the patient with all treatment-related facts, so that the patient can choose); (3) interpretive (the physician, as a counsellor [17], helps the patient to elucidate the preferences and then leaves it to the patient to make a decision); and (4) deliberative (the physician, as a teacher or friend [17], conjointly discusses with the patient the best way forward).

Contemporary medical research advocates the deliberative style [18,19], which can also be referred to as shared decision making [20], as it is thought to consider patients’ values and autonomy and the physician’s caring role better than other interaction styles [17,20]. It was also the preferred interaction style by the majority of patients in preference studies [21,22]. However, there is evidence in the literature that sociodemographic and disease-related variables have an impact on the preferred interaction style. Older patients, for instance, tend to prefer a paternalistic interaction style [23,24], based on the assumption that they are accustomed to physicians being traditionally seen as an authority figure [25]. Among men, there is also a preference for the paternalistic interaction style [23,24]. Fatigue, lacking expertise or knowledge about the condition, and the fear of making a wrong decision are additional reasons mentioned in the literature that explain patients’ preferences for a paternalistic interaction style in case of a severe condition, a newly diagnosed disease, or minor health literacy [26]. No influence of socioeconomic variables has been found [27] that could explain a preference for the informative style over the deliberative style. There seems to be no further evidence in the current literature base that talks about preferences for the interpretive interaction style.

To address these issues, we conducted two studies. The first study aimed to explore if there exist patient preferences for a paternalistic, informative, interpretive, or deliberative interaction style when a CA takes the role of a caregiver. The results of this study informed the second study that explored in more detail (1) which variables moderate preferences for the CA interaction style and (2) whether preferences have an impact on CA-related perceptions (eg, working alliance) and health outcomes (eg, change in health-promoting behaviors). Both studies involved patients diagnosed with chronic obstructive pulmonary disease (COPD), one of the global top four leading causes of premature death from chronic diseases [28].

## Methods

### Study Design

First, we conducted Study 1, a paper-pencil survey with COPD patients treated at a leading Swiss Hospital in the German-speaking part of Switzerland. Besides covering sociodemographic and health-related questions, the survey explored baseline differences in patient preferences for a deliberative, informative, interpretive, or paternalistic interaction style with a hypothetical health care CA.

The outcomes informed Study 2, an online experiment. For this study, we recruited COPD patients from four hospitals in the German-speaking part of Switzerland, from the Swiss Lung Association, and from an honorary led self-help association for COPD patients in the German-speaking part of Switzerland. We designed a between-subject online experiment where patients were randomly assigned to interact with a CA that followed either a deliberative or paternalistic interaction style. We chose these two styles since (1) we have already developed and experimentally tested the implementation of deliberative and paternalistic CA interactions in a recent study [29]; (2) there is the most information in the literature for these two styles regarding the moderating influences of sociodemographic and health-related variables; and (3) we expected to find significant effects when choosing the most and least preferred interaction styles as determined in Study 1. Both studies did not fall within the scope of Human Research Law, according to the local Swiss ethics authority, and thus did not require any formal authorization.

### Sample Size Considerations

The primary objective of Study 1 was to explore whether general differences exist between interaction style preferences of COPD patients for their interaction with a CA. Thus, this part of the study was exploratory by nature and did not contain a detailed power analysis.

We conducted an a priori power analysis for Study 2 using R software (version 3.5.2) and the R package WebPower [30]. To identify a medium effect (f^2^=0.15) [31] in a hierarchical multiple regression with an alpha level of .05, a statistical power of 0.80, a reduced model with one predictor, and a full model with 13 predictors, a total of 127 participants was required.

### Inclusion Criteria

For Study 1, we defined the following inclusion criteria: (1) COPD diagnosis, (2) age of 18 years or older, and (3) ability to speak German.

We defined the same inclusion criteria for Study 2. Here, the first inclusion criterion was checked before distributing the link to the online experiment. The link was only sent out to patients who were registered as COPD patients at any of the participating hospitals, the lung association, or the self-help association. In addition, patients also had to confirm that they have COPD during the online experiment. The second and third inclusion criteria were checked at the beginning of the online experiment. If patients did not confirm either being of age or being able to speak German, the experiment was automatically completed. There were no exclusion criteria.

### Procedure of Study 1 and Study 2

**Figure 1:**
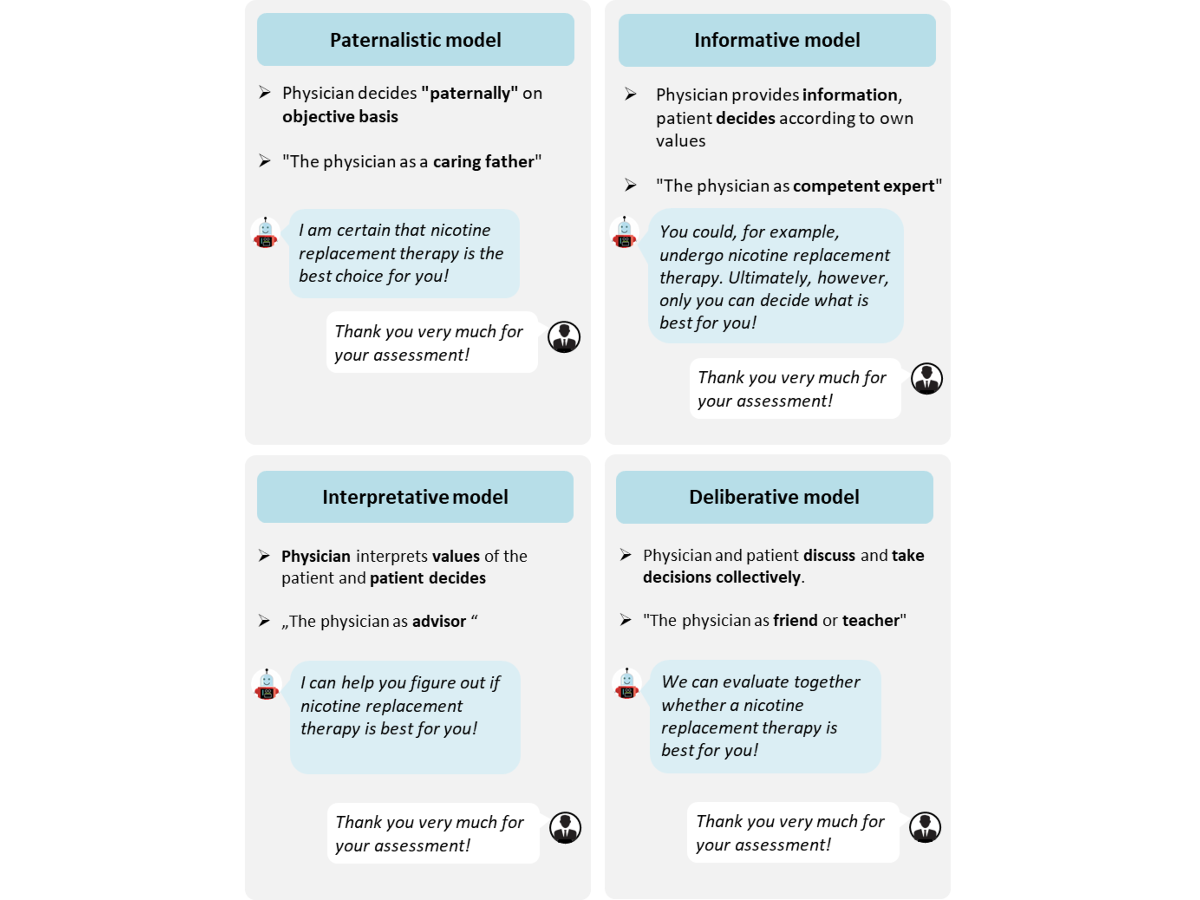
Illustrations outlining the four physician-patient interaction styles.

Study 1 was administered as a paper-and-pencil survey and divided into four parts. Before starting with the actual questions, we provided general information about the survey (ie, we clarified the objective and structure, and provided an illustrative explanation of a CA-based intervention). After querying sociodemographic and health-related questions, the survey explored patients’ preferences regarding their interaction style with a hypothetical CA. Patients could choose from snippets portraying exemplary interactions with a CA, and each snippet depicted a different interaction style (see Figure 1 for an overview).

The procedure of Study 2 was as follows. Participants agreed to the study conditions and confirmed the study inclusion criteria (ie, being of age and being able to speak German). After querying standard demographic data (age, gender, mother tongue, and education), patients answered questions about their general health status and COPD. Patients were then randomly assigned to interact with a CA presenting a deliberative or paternalistic interaction style. The interactions were text-based and followed by a prescripted dialogue based on two predeveloped scripts. These scripts were developed and assessed in a recent study, where they were verified to be perceived as eliciting a deliberative or paternalistic interaction style between a CA and users [29]. During the conversation with the CA, patients could choose between one to three predefined answer options, which were identical in both conditions (ie, deliberative and paternalistic interaction styles). Deviations between the answer options only occurred when needed to keep the conversational flow realistic. Both interactions with the CA (ie, paternalistic and deliberative) were approximately of the same length and duration (38 conversational turns in the deliberative version and 32 in the paternalistic version with a reading duration of roughly over 12 minutes). After the interaction with the CA, participants were asked to evaluate the interaction with the CA on several dimensions. Details about the measures can be found in the measures section below.

To conclude the experiment, participants had to answer a short COPD health literacy quiz. The questions were based on the standardized Bristol COPD Knowledge Questionnaire (BCKQ), a multiple-choice questionnaire developed to measure the disease-specific knowledge of COPD patients [32]. Finally, patients reported on their perception of the length of the study and could leave some free-text feedback. All questions of Study 2 can be found in Multimedia Appendix 1.

### Technical Implementation of CAs for Study 2

We used Qualtrics, a software-based online survey and data collection platform, for the online experiment and for randomly assigning patients to one of the two experimental settings (ie, interaction with a CA using a deliberative interaction style and interaction with a CA using a paternalistic interaction style). We further used Collect.chat, a commercially available chatbot software, to develop the CA dialogues, and iframe to embed the CA into the Qualtrics HTML.

### Recruitment and Management of Study Participants

We recruited the participants for Study 1 from the pulmonology department of a leading Swiss hospital. The patient database included around 1300 patients, and we contacted all by mail. Eligible patients received the printed survey and a letter containing information about the survey and participation conditions. We also provided a prefranked return envelope to reduce the necessary effort to reply to the survey and to minimize financial expenses for the participants. The postal send out took place on April 9 and 10, 2019, and we started to receive responses 1 week later. We received replies for 2 months in total, and the majority of replies reached us in the first 3 weeks.

The patients of Study 2 were recruited during a 3-month period from February to April 2020 at six study sites in Switzerland. The study sites were the pulmonology departments of four hospitals in the German-speaking part of Switzerland, the Swiss Lung Association, and an honorary-led self-help association for COPD patients. Participants were recruited via email or postal mail or in-person by participating health care professionals on-site. When they were recruited in-person, participating health care professionals handed out a flyer to potential participants. This flyer contained some information on the study and a link to the online experiment (see Multimedia Appendix 2 for the flyer). Participating health care professionals had previously received study instructions from the study authors. When patients were recruited via email, they received an email from the participating hospital, the Swiss Lung Association, or the self-help association. In this email, the participating contact person explained the study and asked for voluntary participation. The email further contained the same flyer as used for the on-site recruitment. When participants were recruited via postal mail, they received a letter from the participating hospital with study information and a link to the online experiment, which they had to type into a web browser. The postal letter is presented in Multimedia Appendix 3. We followed a multichannel and multisite recruiting process. More precisely, we contacted 903 patients at three study sites via email and postal mail. Additionally, 110 flyers were printed and displayed at two further study sites. Here, the flyers were displayed in the study sites’ waiting rooms and were free to pick up for the patients. There were no additional prompts by medical personnel to participate in the study. The email and postal mail had the same design and included exactly the same content as the flyer. At the sixth study site, 27 local COPD patient groups were contacted via email. Importantly, the resulting response rate has to be interpreted as a lower bound estimate since patients might have received an invitation more than once. For example, they could have been regular patients in hospital X and, at the same time, also a member of a COPD patient organization that distributed study invitations.

To start the online experiment, participants had to either click on the study link in the email or type the URL in their browser in case they received a postal letter. A simplified URL (ie, only portraying a shorter number of signs) was created with Bitly [33] to access the online experiment and to reduce barriers to participate. The experiment was available online from April to August 2020.

### Measures of Study 1: Paper-and-Pencil Survey

For Study 1, we gathered basic sociodemographic (age, gender, and education) and health-related data (GOLD COPD level) [34]. GOLD stands for Global Initiative for Chronic Obstructive Lung Disease and is an internationally used scale for classifying the severity of COPD [34]. Patients reported sociodemographic and health-related data before being presented with the four main physician-patient interaction styles.

#### Sociodemographic Data

For the item *age*, patients reported their birth year. To derive patients’ actual age, their birth year was subtracted from 2020 in the descriptive analyses. For the item *gender*, patients could choose their gender from a tick box with answer options “female,” “male,” and “no answer.” For the item *education*, patients could choose one of the following four different options: *no formal education*, *secondary 1 education*, *secondary 2 education*, and *tertiary education.* Drawing on the 2011 International Standard Classification of Education (ISCED) Scheme [35], a comprehensive framework for comparing and organizing education programs and qualification across countries, these options translate to other global educational systems, such as the US system as follows. *Secondary 1 education* in Switzerland corresponds to level 2 of the ISCED scheme (ISCED 2, lower secondary education) and 9 years of education completed. ISCED 2 translates to completing junior high school in the United States. *Secondary 2 education* corresponds to level 3 of the ISCED scheme (ISCED 3, upper secondary education) and 13 years of education completed. ISCED 3 translates to completing a senior high school degree in the United States. *Tertiary education* in Switzerland (ISCED 6-8) encompasses bachelor’s, master’s, and PhD degrees, corresponding to a total of 16, 18, and 21 years needed to complete these educational levels (equivalent to the degrees in the United States) [36,37].

#### Health-Related Data

We queried the *GOLD COPD level* for each patient with the item “What is your GOLD rating?” (translation by the authors) on a 5-point scale ranging from 1 (GOLD 1) to 5 (I do not know). The highest and most severe GOLD level is GOLD 4.

Next, we introduced the four main physician-patient interaction styles as per a previous report [17], and patients were asked which of the four they would prefer for their interaction with a CA. We provided a definition of the respective role of the physician in the interaction style.

### Measures of Study 2: Online Experiment

Study 2 was pretested with two pulmonologists of a leading Swiss hospital (both specialized in COPD), one advanced practitioner nurse in lung disease, and 10 PhD students of the Swiss Federal Institute of Technology in Zurich. We aimed to identify possible problems in terms of clarity, accuracy, and relevance for assessing health-related measures that are specific for COPD. Based on the feedback, a few changes were made to improve the wording of the questions and the order of the items.

For the main analyses and for assessing the various aspects of the two interaction styles, we gathered basic sociodemographic data (age, gender, and education), health-related data (GOLD COPD level, years since COPD diagnosis, and disease literacy), and interaction-related data (willingness to change, relationship quality, intention to continue interacting, and working alliance).

#### Sociodemographic and Health-Related Data

Sociodemographic data were gathered before the interaction with the CA. For the item *age*, patients could choose their birth year from a dropdown menu, ranging from 1900 to 2020. To derive patients’ age, their birth year was subtracted from 2020 in the analyses. For the item *gender*, patients could choose their gender from a tick box with answer options “female,” “male,” and “no answer.” For the item *education*, patients could choose from 12 different options from a dropdown menu (eg, “apprenticeship” [translated by the authors]; all original German measures can be found in Multimedia Appendix 4). For the analyses, educational attainment was recoded as years of education based on the 2011 ISCED scheme [35].

Health-related data were gathered before the interaction with the CA. Just as in Study 1, we first queried the *GOLD COPD level* for each patient with the item “What is your GOLD rating?” (translation by the authors) on a 5-point scale ranging from 1 (GOLD 1) to 5 (I do not know). The highest and most severe GOLD level is GOLD 4. Second, we measured *years since COPD diagnosis* with the item “Have you been diagnosed with COPD?” (translation by the authors), with answer options “yes” and “no” to ensure that patients confirm their COPD disease, and the item “If so, in which year?” (translation by the authors), with a dropdown menu to select a year between 1900 and 2020. To derive patients’ years of experience with the disease, the diagnosis year was subtracted from 2020 in the analyses. Third, we measured *disease literacy*. Disease literacy was assessed using the BCKQ [32]. To keep the handling time for patients as short as possible, we selected 10 items of the BCKQ after consultation with two nurses who frequently use the questionnaire themselves with their patients. For example, patients had to mark the statement “COPD is commonly an inherited disease” (translation by the authors) as “true,” “false,” or “I do not know.” If they evaluated the statement correctly, they were given 1 point, and in case of a false or “I do not know” answer, they were given 0 points. We built a sum score over the 10 items, with higher values indicating higher disease literacy. The complete BCKQ (68 items) was reported to have reasonable internal consistency with a Cronbach alpha of .73 in previous studies [32]. The 10-item subset used in our study had a Cronbach alpha of .62, which, though not ideal, can still be considered acceptable [38], especially given the drastically reduced number of items.

#### Interaction-Related Data

Interaction-related data were gathered after the interaction with the CA Robo. We first measured *willingness to change* with the item “Was Robo able to motivate you for the proposed exercise?” (translation by the authors), with answer options “yes” and “no.” This item was adapted from a previous report [39]. We further measured *relationship quality* with the German adapted item [40] “How would you characterize your relationship with Robo?” (translation by the authors), with answers on a scale ranging from 1 (“complete stranger”) to 5 (“close friend”), and the German adapted item [41] “I think Robo liked me” (translation by the authors), with answers fixed on a 5-point Likert scale ranging from 1 (“not at all”) to 5 (“very much”). Next, we measured *intention to continue interacting* with the German adapted item [42] “I would like to continue using Robo” (translation by the authors), with answers fixed on a 6-point Likert scale ranging from 1 (“strongly disagree”) to 6 (“strongly agree”). Finally, we measured *working alliance* between the patients and the CA Robo with a German-adapted version of the Working Alliance Inventory-Short Revised (WAI-SR) [43]. Here, we measured the two subscales *attachment bond* and *goal agreement* (eg, “Robo and I respect each other,” translation by the authors), with answers fixed on a 5-point Likert scale ranging from 1 (“rarely”) to 5 (“always”) (Multimedia Appendix 4). We decided to omit measuring the third subscale *task agreement* as it does not directly relate to our research questions. For secondary analyses (Multimedia Appendix 5), we measured additional interaction-related data (eg, satisfaction with the interaction and recommendation to a friend).

## Results

### Results of Study 1: Paper-and-Pencil Survey

Preferences for the paternalistic, informative, deliberative, or interpretive style are depicted by gender, age, educational levels, and disease severity. Out of 181 participants who started the survey, only 139 participants who completed the CA preference task were included in the final sample of Study 1. Moreover, 22 additional participants were excluded because they reported not having COPD. The final sample consisted of 117 participants with a mean age of 65.7 years and a mean GOLD classification value of 2.87. Of the 117 participants, 66 were male. Descriptive statistics can be found in Table 1. R scripts for all tables can be found in Multimedia Appendix 6. Missing data were dealt with by list-wise deletion because of the small number of participants having missing values for a variable of interest.

**Table 1:** Descriptive statistics of the participants in Study 1 (N=117).

Characteristic		Value
**Gender, n (%)**		
	Male	66 (56%)
	Female	51 (44%)
Age (years), mean (SD)		65.67 (10.92)
**Education, n (%)**		
	None	7 (6%)
	Secondary I	15 (13%)
	Secondary II	56 (48%)
	Tertiary	36 (31%)
COPD^a^ severity value, mean (SD)		2.87 (0.98)
**COPD severity, n (%)**		
	GOLD^b^ 1	7 (6%)
	GOLD 2	20 (17%)
	GOLD 3	24 (21%)
	GOLD 4	24 (21%)
	Do not know	35 (30%)

^a^COPD: chronic obstructive pulmonary disease.

^b^GOLD: Global Initiative for Chronic Obstructive Lung Disease.

#### Gender

Across CA categories, women most often chose the deliberative CA type, whereas men preferred the informative and deliberative CA types (26 vs 24; Table 2). Within each category of the deliberative, paternalistic, or interpretive CA type, men and women were fairly equally represented. Men constituted 77% (26/34 persons) of persons who preferred the informative CA type.

**Table 2:** Conversational agent preferences by gender.

Variable (gender)	Value, n^a^ (%^b^ by conversational agent category)				Sum (N=117), n
	Paternalistic (n=7)	Informative (n=34)	Interpretive (n=26)	Deliberative (n=50)	
Male	4 (57%)	26 (77%)	12 (46%)	24 (48%)	66
Female	3 (43%)	8 (24%)	14 (54%)	26 (52%)	51

^a^Numbers represent the absolute numbers of participants.

^b^Percentages of male/female participants present in each conversational agent category are given in parentheses.

#### Age

Younger participants (40-50 years old) preferred the deliberative CA type over the paternalistic CA type. Participants in the age group 51-60 years preferred the informative type. Participants in the age groups 61-70 and 71-80 years most often chose the deliberative type, and the oldest participants (81-90 years old) were fairly equally distributed across the informative, interpretive, and deliberative CA types. Within categories, 57% (4/7) of participants who chose the paternalistic CA type were in the youngest age category (40-50 years old). On the other hand, in the interpretive and deliberative groups, 78% (20/26) and 70% (35/50) of participants, respectively, were older than 60 years (Table 3).

**Table 3:** Conversational agent preferences by age.

Variable (age)	Value, n^a^ (%^b^ by conversational agent category)				Sum (N=117), n
	Paternalistic (n=7)	Informative (n=34)	Interpretive (n=26)	Deliberative (n=50)	
40-50 years	4 (57%)	1 (3%)	1 (4%)	6 (12%)	12
51-60 years	2 (29%)	12 (35%)	5 (19%)	9 (18%)	28
61-70 years	0 (0%)	9 (27%)	9 (35%)	14 (28%)	32
71-80 years	1 (14%)	9 (27%)	9 (35%)	19 (38%)	38
81-90 years	0 (0%)	3 (9%)	2 (8%)	2 (4%)	7

^a^Numbers represent the absolute numbers of participants.

^b^Percentages of age category participants present in each conversational agent category are given in parentheses.

#### Educational Levels

Participants without any formal education preferred the informative CA type. Participants who finished secondary I were fairly equally distributed across categories. Participants with higher educational levels (secondary II and tertiary) preferred the deliberative CA type (Table 4). This pattern was more distinct for secondary II than tertiary, where participants also often chose the informative CA type. Within categories, participants who had secondary II and tertiary education constituted 85% (40/47) of those who chose the deliberative type. Similarly, participants who had secondary II and tertiary education constituted 87% (23/26) of those who preferred the interpretive type. In the paternalistic condition, only the first three educational levels were represented (no formal education, secondary I, and secondary II).

**Table 4:** Conversational agent preferences by educational level.

Variable (education)	Value, n^a^ (%^b^ by conversational agent category)				Sum (N=114), n
	Paternalistic (n=7)	Informative (n=34)	Interpretive (n=26)	Deliberative (n=47)	
Secondary I	3 (43%)	4 (12%)	3 (12%)	5 (11%)	15
Secondary II	3 (43%)	14 (41%)	14 (54%)	25 (53%)	56
Tertiary	0 (0%)	12 (35%)	9 (35%)	15 (32%)	36
No formal education	1 (14%)	4 (12%)	0 (0%)	2 (4%)	7

^a^Numbers represent the absolute numbers of participants.

^b^Percentages of educational level participants present in each conversational agent category are given in parentheses.

#### Severity of COPD

Across CA categories, participants with less severe disease (GOLD 1) preferred the informative and interpretive CA types. Participants with mid-severe disease (GOLD 2 and 3) preferred the deliberative CA type. Participants with severe disease (GOLD 4) preferred the deliberative and informative CA types. Within categories, participants with higher disease levels (GOLD 3+4) constituted 100% (3/3) of those who preferred the paternalistic CA type (Table 5).

**Table 5:** Conversational agent preferences by Global Initiative for Chronic Obstructive Lung Disease classification.

Variable (GOLD^a^ classification)	Value, n^b^ (%^c^ by conversational agent category)^d^				Sum (N=75), n
	Paternalistic (n=3)	Informative (n=27)	Interpretive (n=12)	Deliberative (n=33)	
GOLD 1	0 (0%)	3 (11%)	3 (25%)	1 (3%)	7
GOLD 2	0 (0%)	7 (26%)	4 (33%)	9 (27%)	20
GOLD 3	2 (67%)	6 (22%)	3 (25%)	13 (39%)	24
GOLD 4	1 (33%)	11 (41%)	2 (17%)	10 (30%)	24

^a^GOLD: Global Initiative for Chronic Obstructive Lung Disease.

^b^Numbers represent absolute numbers of participants.

^c^Percentages of GOLD classification category participants present in each conversational agent category are given in parentheses.

^d^The data of a maximum of seven out of 117 participants were deleted for Table 5 because they did not report any information on their GOLD classification (neither “GOLD 1-4” nor “I do not know”).

### Results of Study 2: Online Experiment

We conducted hierarchical multiple regression modeling to predict participants’ self-reported interaction quality with the CA, that is, *willingness to change*, *relationship quality*, *intention to continue interaction*, and *working alliance*, based on (1) the type of CA (paternalistic/deliberative), (2) patients’ demographics (age, gender, and education), and (3) COPD-related measures (GOLD, COPD disease literacy, and experience with COPD). Each outcome was predicted in a three-step procedure. The first block added to the model was the CA type (labelled “model 1”). The second block contained the CA type and participants’ demographics (labelled “model 2”), and the third block consisted of the CA type, participants’ demographics, and COPD-related measures (labelled “model 3”). *Relationship quality*, *intention to continue interaction,* and *working alliance* were measured on a metric scale. We calculated hierarchical linear regressions for those outcomes and logistic regression for the binary outcome *willingness to change*. As in Study 1, missing data were dealt with by list-wise deletion.

#### Analysis of Sociodemographic and Health-Related Data

The descriptive statistics of the experiment are shown in Table 6. Out of 168 participants who started the survey, 124 completed the survey. One additional participant was excluded because of age (<18 years old), leading to a final sample of 123 participants. Of those 123 participants, 76 were male, with a mean age of 67.8 years and a mean duration of 8.4 years since their COPD diagnosis. The mean GOLD classification value was 2.70.

**Table 6:** Descriptive statistics of the participants in Study 2 (N=123).

Characteristic		Value
**Gender, n (%)**		
	Male	76 (62%)
	Female	47 (38%)
Age (years), mean (SD)		67.82 (9.37)
Education (years of formal education), mean (SD)		14.28 (2.37)
COPD^a^ severity value, mean (SD)		2.70 (0.88)
**COPD severity, n (%)**		
	GOLD^b^ 1	6 (5%)
	GOLD 2	24 (20%)
	GOLD 3	29 (24%)
	GOLD 4	14 (11%)
	Do not know	35 (28%)

^a^COPD: chronic obstructive pulmonary disease.

^b^GOLD: Global Initiative for Chronic Obstructive Lung Disease.

#### Analyses of Interaction-Related Data

For our analysis, we introduce interaction-related outcomes, defined in terms of the outcome variables *willingness to change*, *relationship quality*, *intention to continue interaction*, and two dimensions of *working alliance* (*attachment bond* and *goal agreement*). Better interaction-related outcomes indicate a higher willingness to change one’s behavior after interaction with the CA, a higher perceived relationship quality, a higher motivation to continue interacting with the CA, and a higher-rated reported working alliance with the CA in terms of perceived close attachment bond and common goal agreement. Multimedia Appendix 5 presents the results of additional interaction-related data (eg, satisfaction with the interaction and recommendation to a friend). The R scripts for all analyses are presented in Multimedia Appendix 7.

#### Willingness to Change

Overall, participants who interacted with a paternalistic CA reported being more willing to change their behavior based on the CA intervention than those who worked with a deliberative CA (Table 7). There were no substantial interaction effects between CA type and participants’ demographics or CA type and patients’ COPD-related characteristics.

**Table 7:** Regression of conversational agent type, participants’ demographics, and chronic obstructive pulmonary disease–related characteristics in terms of participants’ willingness to change their behavior after conversational agent interaction.

Variable	Model 1^a^, regression coefficient (95% CI)	Model 2^b^, regression coefficient (95% CI)	Model 3^c^, regression coefficient (95% CI)
Intercept	0.833 (0.724 to 0.943)	0.840 (0.699 to 0.980)	0.840 (0.640 to 1.039)
CA^d^ type^e^	−0.183 (−0.338 to −0.029)^f^	−0.179 (−0.388 to 0.030)	−0.074 (−0.350 to 0.203)
Gender^g^	N/A^h^	−0.024 (−0.283 to 0.235)	0.041 (−0.325 to 0.406)
Age	N/A	−0.037 (−0.161 to 0.088)	−0.010 (−0.207 to 0.188)
Education^i^	N/A	−0.108 (−0.229 to 0.012)	−0.064 (−0.231 to 0.103)
Gender*CA type	N/A	−0.035 (−0.387 to 0.317)	−0.163 (−0.627 to 0.301)
Age*CA type	N/A	0.003 (−0.162 to 0.169)	−0.145 (−0.386 to 0.095)
Education*CA type	N/A	0.091 (−0.078 to 0.259)	0.055 (−0.157 to 0.268)
GOLD^j^	N/A	N/A	0.053 (−0.105 to 0.210)
COPD^k^ literacy	N/A	N/A	0.165 (−0.047 to 0.377)
Experience^l^	N/A	N/A	−0.127 (−0.294 to 0.040)
GOLD*CA type	N/A	N/A	−0.040 (−0.261 to 0.181)
COPD literacy*CA type	N/A	N/A	−0.058 (−0.311 to 0.194)
Experience*CA type	N/A	N/A	0.152 (−0.078 to 0.381)

^a^Model 1 includes the CA type. It has 120 observations and an Aikaike Information Criterion value of 142.880 (a smaller value is associated with better model fit).

^b^Model 2 includes the CA type and participants’ demographics. It has 113 observations and an Aikaike Information Criterion value of 146.953 (a smaller value is associated with better model fit).

^c^Model 3 includes the CA type, participants’ demographics, and COPD-related measures. It has 67 observations and an Aikaike Information Criterion value of 83.496 (a smaller value is associated with better model fit).

^d^CA: conversational agent.

^e^CA type is coded as follows: 0=paternalistic, 1=deliberative.

^f^Significant (*P*<.05).

^g^Gender is coded as follows: 0=male, 1=female.

^h^N/A: not applicable.

^i^Education is measured in years of formal education.

^j^GOLD: Global Initiative for Chronic Obstructive Lung Disease.

^k^COPD: chronic obstructive pulmonary disease.

^l^Experience with COPD in years since COPD diagnosis.

#### Relationship Quality

On average, older participants reported better relationship quality with the CA than younger participants, irrespective of the CA type. Participants with more severe COPD reported better relationship quality with the CA than participants with less severe COPD, irrespective of the CA type. There was a negative interaction effect between CA type and age, implying that older participants preferred a paternalistic CA and younger participants preferred a deliberative CA with respect to relationship quality (Table 8).

**Table 8:** Regression of conversational agent type, participants’ demographics, and chronic obstructive pulmonary disease–related characteristics in terms of participants’ relationship quality with the conversational agent.

Variable	Model 1^a^, regression coefficient (95% CI)	Model 2^b^, regression coefficient (95% CI)	Model 3^c^, regression coefficient (95% CI)
Intercept	−0.073 (−0.326 to 0.181)	−0.030 (−0.350 to 0.290)	−0.284 (−0.774 to 0.206)
CA^d^ type^e^	0.146 (−0.213 to 0.504)	−0.007 (−0.482 to 0.469)	0.176 (−0.504 to 0.857)
Gender^f^	N/A^g^	−0.399 (−0.988 to 0.191)	0.262 (−0.637 to 1.161)
Age	N/A	0.155 (−0.129 to 0.438)	0.615 (0.129 to 1.100)^h^
Education^i^	N/A	−0.031 (−0.306 to 0.243)	0.240 (−0.171 to 0.652)
Gender*CA type	N/A	0.546 (−0.255 to 1.347)	−0.377 (−1.519 to 0.764)
Age*CA type	N/A	−0.215 (−0.592 to 0.162)	−0.774 (−1.366 to −0.181)^h^
Education*CA type	N/A	0.057 (−0.326 to 0.440)	−0.018 (−0.541 to 0.504)
GOLD^j^	N/A	N/A	0.398 (0.011 to 0.786)^h^
COPD^k^ literacy	N/A	N/A	0.324 (−0.198 to 0.846)
Experience^l^	N/A	N/A	−0.220 (−0.631 to 0.191)
GOLD*CA type	N/A	N/A	−0.132 (−0.675 to 0.411)
COPD literacy*CA Type	N/A	N/A	−0.031 (−0.652 to 0.590)
Experience*CA type	N/A	N/A	0.194 (−0.370 to 0.759)

^a^Model 1 includes the CA type. It has 120 observations and an R^2^ value of 0.005.

^b^Model 2 includes the CA type and participants’ demographics. It has 113 observations and an R^2^ value of 0.039.

^c^Model 3 includes the CA type, participants’ demographics, and COPD-related measures. It has 67 observations and an R^2^ value of 0.283.

^d^CA: conversational agent.

^e^CA type is coded as follows: 0=paternalistic, 1=deliberative.

^f^Gender is coded as follows: 0=male, 1=female.

^g^N/A: not applicable.

^h^Significant (*P*<.05).

^i^Education is measured in years of formal education.

^j^GOLD: Global Initiative for Chronic Obstructive Lung Disease.

^k^COPD: chronic obstructive pulmonary disease.

^l^Experience with COPD in years since COPD diagnosis.

#### Intention to Continue Interaction

Disease severity positively predicted participants’ intention to continue interacting with the CA after the interaction ended. The higher a participant’s GOLD classification, the higher was his or her intention to continue (Table 9). Participants with fewer years of experience with COPD reported a higher intention to continue the interaction, irrespective of the assigned CA type. Older participants reported being more likely to continue the CA interaction when working with a paternalistic CA, and younger participants reported that when working with a deliberative CA.

**Table 9:** Regression of conversational agent type, participants’ demographics, and chronic obstructive pulmonary disease–related characteristics in terms of participants’ intention to continue interacting with the conversational agent.

Variable	Model 1^a^, regression coefficient (95% CI)	Model 2^b^, regression coefficient (95% CI)	Model 3^c^, regression coefficient (95% CI)
Intercept	0.112 (−0.140 to 0.365)	0.046 (−0.277 to 0.369)	0.134 (−0.263 to 0.531)
CA^d^ type^e^	−0.224 (−0.581 to 0.133)	−0.169 (−0.649 to 0.311)	−0.279 (−0.831 to 0.272)
Gender^f^	N/A^g^	−0.037 (−0.632 to 0.558)	0.404 (−0.325 to 1.133)
Age	N/A	−0.075 (−0.361 to 0.211)	0.178 (−0.216 to 0.572)
Education^h^	N/A	0.053 (−0.224 to 0.331)	0.019 (−0.314 to 0.353)
Gender*CA type	N/A	−0.011 (−0.819 to 0.797)	−0.637 (−1.563 to 0.288)
Age*CA type	N/A	−0.063 (−0.444 to 0.317)	−0.485 (−0.965 to −0.005)^i^
Education*CA type	N/A	−0.162 (−0.549 to 0.224)	−0.084 (−0.507 to 0.340)
GOLD^j^	N/A	N/A	0.420 (0.106 to 0.734)^i^
COPD^k^ literacy	N/A	N/A	0.199 (−0.224 to 0.622)
Experience^l^	N/A	N/A	−0.391 (−0.724 to −0.058)^i^
GOLD*CA type	N/A	N/A	−0.153 (−0.593 to 0.287)
COPD literacy*CA type	N/A	N/A	0.277 (−0.226 to 0.781)
Experience*CA type	N/A	N/A	0.277 (−0.181 to 0.735)

^a^Model 1 includes the CA type. It has 120 observations and an R^2^ value of 0.013.

^b^Model 2 includes the CA type and participants’ demographics. It has 113 observations and an R^2^ value of 0.026.

^c^Model 3 includes the CA type, participants’ demographics, and COPD-related measures. It has 67 observations and an R^2^ value of 0.411.

^d^CA: conversational agent.

^e^CA type is coded as follows: 0=paternalistic, 1=deliberative.

^f^Gender is coded as follows: 0=male, 1=female.

^g^N/A: not applicable.

^h^Education is measured in years of formal education.

^i^Significant (*P*<.05).

^j^GOLD: Global Initiative for Chronic Obstructive Lung Disease.

^k^COPD: chronic obstructive pulmonary disease.

^l^Experience with COPD in years since COPD diagnosis.

#### Working Alliance (Attachment Bond)

We found a substantial negative interaction effect among age, CA type, and reported attachment bond with the CA. This indicates that older participants had a higher attachment bond when working with the paternalistic CA type and younger participants had that when working with the deliberative CA type. Overall, participants who had a higher disease literacy of COPD also reported better attachment bond (Table 10), irrespective of the assigned CA type.

**Table 10:** Regression of conversational agent type, participants’ demographics, and chronic obstructive pulmonary disease–related characteristics in terms of participants’ working alliance (attachment bond) with the conversational agent.

Variable	Model 1^a^, regression coefficient (95% CI)	Model 2^b^, regression coefficient (95% CI)	Model 3^c^, regression coefficient (95% CI)
Intercept	−0.123 (−0.375 to 0.129)	−0.161 (−0.482 to 0.161)	−0.424 (−0.932 to 0.083)
CA^d^ type^e^	0.245 (−0.111 to 0.602)	0.339 (−0.138 to 0.817)	0.526 (−0.178 to 1.231)
Gender^f^	N/A^g^	0.001 (−0.591 to 0.593)	0.721 (−0.210 to 1.651)
Age	N/A	0.141 (−0.144 to 0.425)	0.302 (−0.200 to 0.805)
Education^h^	N/A	−0.094 (−0.370 to 0.181)	0.061 (−0.365 to 0.487)
Gender*CA type	N/A	−0.186 (−0.990 to 0.619)	−0.939 (−2.121 to 0.242)
Age*CA type	N/A	−0.284 (−0.662 to 0.095)	−0.650 (−1.263 to −0.037)^i^
Education*CA type	N/A	0.047 (−0.338 to 0.432)	−0.022 (−0.563 to 0.518)
GOLD^j^	N/A	N/A	0.278 (−0.123 to 0.679)
COPD^k^ literacy	N/A	N/A	0.595 (0.055 to 1.135)^i^
Experience^l^	N/A	N/A	−0.307 (−0.732 to 0.118)
GOLD*CA type	N/A	N/A	−0.420 (−0.982 to 0.142)
COPD literacy*CA type	N/A	N/A	−0.405 (−1.048 to 0.237)
Experience*CA type	N/A	N/A	0.537 (−0.047 to 1.122)

^a^Model 1 includes the CA type. It has 120 observations and an R^2^ value of 0.015.

^b^Model 2 includes the CA type and participants’ demographics. It has 113 observations and an R^2^ value of 0.044.

^c^Model 3 includes the CA type, participants’ demographics, and COPD-related measures. It has 67 observations and an R^2^ value of 0.221.

^d^CA: conversational agent.

^e^CA type is coded as follows: 0=paternalistic, 1=deliberative.

^f^Gender is coded as follows: 0=male, 1=female.

^g^N/A: not applicable.

^h^Education is measured in years of formal education.

^i^Significant (*P*<.05).

^j^GOLD: Global Initiative for Chronic Obstructive Lung Disease.

^k^COPD: chronic obstructive pulmonary disease.

^l^Experience with COPD in years since COPD diagnosis.

#### Working Alliance (Goal Agreement)

Irrespective of the CA type participants were working with, those with a higher disease literacy reported higher perceived support to achieve their goals by the CA. Participants with fewer years of experience with COPD reported higher perceived support to achieve their goals by the CA. Older participants reported higher support by the CA when in the paternalistic condition, and younger participants reported that when in the deliberative condition. A positive interaction effect between CA type and experience with COPD implied that participants who were more experienced with COPD reported better perceived support in achieving their goals when interacting with a deliberative CA and participants who were less experienced with COPD reported that when interacting with a paternalistic CA (Table 11).

**Table 11:** Regression of conversational agent type, participants’ demographics, and chronic obstructive pulmonary disease–related characteristics in terms of participants’ working alliance (goal agreement) with the conversational agent.

Variable	Model 1^a^, regression coefficient (95% CI)	Model 2^b^, regression coefficient (95% CI)	Model 3^c^, regression coefficient (95% CI)
Intercept	0.017 (−0.237 to 0.271)	0.001 (−0.322 to 0.323)	−0.101 (−0.585 to 0.383)
CA^d^ type^e^	−0.034 (−0.394 to 0.325)	0.120 (−0.358 to 0.599)	0.219 (−0.453 to 0.891)
Gender^f^	N/A^g^	−0.066 (−0.660 to 0.528)	0.515 (−0.373 to 1.402)
Age	N/A	−0.000 (−0.286 to 0.285)	0.247 (−0.233 to 0.726)
Education^h^	N/A	−0.126 (−0.402 to 0.151)	−0.201 (−0.608 to 0.206)
Gender*CA type	N/A	−0.256 (−1.063 to 0.551)	−0.840 (−1.967 to 0.287)
Age*CA type	N/A	−0.134 (−0.514 to 0.245)	−0.592 (−1.176 to −0.007)^i^
Education*CA type	N/A	0.158 (−0.228 to 0.544)	0.233 (−0.283 to 0.748)
GOLD^j^	N/A	N/A	0.163 (−0.219 to 0.546)
COPD^k^ literacy	N/A	N/A	0.690 (0.175 to 1.205)^i^
Experience^l^	N/A	N/A	−0.435 (−0.841 to −0.029)^i^
GOLD*CA type	N/A	N/A	−0.324 (−0.860 to 0.212)
COPD literacy*CA type	N/A	N/A	−0.384 (−0.997 to 0.229)
Experience*CA type	N/A	N/A	0.570 (0.012 to 1.128)^i^

^a^Model 1 includes the CA type. It has 120 observations and an R^2^ value of 0.0003.

^b^Model 2 includes the CA type and participants’ demographics. It has 113 observations and an R^2^ value of 0.030.

^c^Model 3 includes the CA type, participants’ demographics, and COPD-related measures. It has 67 observations and an R^2^ value of 0.273.

^d^CA: conversational agent.

^e^CA type is coded as follows: 0=paternalistic, 1=deliberative.

^f^Gender is coded as follows: 0=male, 1=female.

^g^N/A: not applicable.

^h^Education is measured in years of formal education.

^i^Significant (*P*<.05).

^j^GOLD: Global Initiative for Chronic Obstructive Lung Disease.

^k^COPD: chronic obstructive pulmonary disease.

^l^Experience with COPD in years since COPD diagnosis.

### Summary

In summary, we found evidence that age and experience with COPD inform participants' preferences for a deliberative or paternalistic interaction style of the CA. Older participants reported better interaction-related outcomes when interacting with a paternalistic CA, whereas younger participants reported that when interacting with a deliberative CA. Participants with fewer years of personal experience with COPD reported better interaction-related outcomes when interacting with a paternalistic CA, whereas those with more years of personal experience reported that when interacting with a deliberative CA. We did not find evidence for gender, disease level, and disease literacy. Irrespective of the CA type, disease literacy positively predicted both dimensions of working alliance, and participants with fewer years of experience with COPD reported higher perceived support in goal agreement by the CA and were more motivated to continue the interaction with the CA. A more severe disease level was associated with higher motivation to continue the interaction with the CA. Participants who worked with a paternalistic CA were more likely to change their behavior based on the intervention. Thus, our results indicate that knowing the age and years of experience of a patient with COPD can help to decide which interaction style to choose for the patient in order to increase interaction-related outcomes for the patient.

## Discussion

### Principal Findings

In this work, we investigated the preferences of patients with COPD for specific interaction styles of health care CAs. The interaction style between health care professionals and patients has long been recognized as a key success factor for chronic disease management and final treatment success [44,45]. Given the rising number of chronically diseased patients and the associated financial and personal burdens, CAs represent scalable and ubiquitous digital tools to support chronic patients and relieve human health care professionals. A systematic approach for inducing two specific interaction styles into CAs in a health care setting has previously been developed and validated [29].

In our first study, we determined baseline differences for preferred interaction styles between 117 COPD patients and CAs. We showed that differences in preferences for specific interaction styles for the interaction between chronically diseased patients and CAs exist. In our second study, we explored the patterns of preferences for two specific interaction styles in 123 COPD patients. We found evidence that younger patients reported better interaction-related outcomes when interacting with a deliberative CA, while older COPD patients reported better interaction-related outcomes when interacting with a paternalistic CA. Additionally, COPD patients with longer personal experience with the disease reported better interaction-related outcomes when interacting with a deliberative CA. Moreover, COPD patients with lower COPD disease literacy reported better interaction-related outcomes when interacting with a paternalistic CA. Gender, disease severity, or disease literacy did not affect any preferences for specific interaction styles. Nevertheless, we found evidence that disease literacy, in general, positively predicted both dimensions of working alliance independent of the interaction style.

This paper is especially important for the development of personalized CAs in the context of digital health care, with a focus on chronic diseases. To our knowledge, this is the first investigation that systematically evaluated the preferences of chronic patients for their interaction style with CAs. While CAs have primarily been developed portraying a single interaction style for every human counterpart interacting with them, medical research has long stated the crucial importance of deploying personal interaction styles in order to improve patient satisfaction [40], treatment adherence, and final treatment outcome [46,47]. Addressing the gap in the literature regarding differentiated and personalized interaction styles for patient-CA interactions and adding to the growing body of literature on CA personalization [10,11], this paper now provides the first evidence that chronic patients report better interaction-related outcomes when interacting with CAs that display personalized interaction styles.

The findings of these two studies further inform the pairing of chronic patients to CAs that are personalized at the level of their interaction style. While medical research postulates the relevance of five factors (gender [23,24], age [23,24], disease level [26,48], personal experience with a disease [49], and disease literacy [50,51]) that influence the patient-physician interaction, we showed that not all of these aspects are similarly important when it comes to coupling chronic patients with CAs. Our first results indicate that knowing the age and personal disease experience of the patient is sufficient to decide which interaction style results in increased interaction-related outcomes for the patient at hand. While these are the first results from a restricted sample in an experimental setting, the implications for CA deployment could be significant. Especially from a privacy perspective, the findings would reduce the amount of personal patient data needed to achieve an advantageous CA-patient allocation, as only these two data points can be gathered instead of obtaining a whole plethora of personal data. In addition, these two data points can be easily collected at the start of the patient-CA interaction, without any specific (medical) knowledge needed for assessing them. This could reduce the work of health care professionals, whose time is limited and costly, as the best-fitting CA could be allocated based on responses to a simple digital questionnaire at the beginning of the patient-CA interaction. Notwithstanding these potential possibilities, more research needs to be done to be able to robustly understand patients’ preferences in detail so that industry applications can be developed and reliably used.

### Strengths and Limitations

This paper has several strengths. First, we followed a two-step approach by determining baseline differences of COPD patients’ preferences for their interaction with a CA in Study 1 and subsequently expanding the findings to Study 2, a between-subject online experiment. Second, we deployed a systematic and validated approach for inducing two specific interaction styles into the patient-CA interaction [29]. Third, we continuously ensured an objective approach by integrating both theoretical knowledge and applied medical expertise into the development of the experiment. We did this by closely collaborating with medical professionals. Here, we worked together with not only medical experts on COPD (the chronic disease subject of this paper), but also health care professionals from other fields to reduce the risk of bias. In addition, we integrated the views of both senior and novice health care professionals to reflect traditional paternalistic-based training and current shared decision making–based training. Fourth, we focused on investigating the preferences of a specific target population, that is, patients with COPD. This focus on a relatively homogeneous patient group allowed us to delve into depth and gain a profound understanding of their preferences.

This work also has limitations. First, we tested baseline differences between all four major interaction styles in Study 1 (a paper-pencil study), but we only tested personal preferences for the deliberative and paternalistic interaction styles in Study 2 (online experiment). These are the two interaction styles where a systematic and validated approach for inducing the specific interaction style into the CA-patient interaction for a digital online setting exists. They further represent the start point and endpoint of a hypothetical ethical development process of a model patient-physician interaction [52]. Nevertheless, the results from Study 1 indicated that some patients might have personal preferences for other interaction styles than these two. The preferences for these interaction styles need to be investigated by future research. In addition, we could see differences in the preference allocation between the studies. While older participants in Study 2 preferred a paternalistic CA when evaluating relationship quality, 57% (4/7) of participants who chose the paternalistic style in Study 1 were in the youngest of the tested age ranges. This difference could be assumed to have originated in the respective study setup. Participants could choose from one single interaction snippet of the four main different interaction styles, and participants in Study 2 had a real interaction with a CA, but only the deliberative or paternalistic CA. As such, further research is needed to robustly understand patients’ preferences, especially when it comes to a real interaction with a CA. Second, the study population only included German-speaking patients based in one country. It could be that other languages or regions influence different interaction style preferences and personal requirements. Third, disease-related patient inputs (eg, GOLD status and years of diagnosis) were self-reported and hence not verifiable. Additionally, not all patients reported their GOLD status. Fourth, we only modeled the first part of an initial interaction between a patient and a CA. In reality, patients would need to interact over a prolonged period of time when a CA supports their chronic disease management. Fifth, we used a paper-based snippet of a hypothetical patient-CA interaction in Study 1 and a prescripted and rule-based CA in Study 2. While these two approaches were necessary because of the study condition in Study 1 (we sent a physical letter to the patients) and to control the experimental condition in Study 2, both approaches have their limitations when it comes to emulating a naturalistic patient-physician interaction. We are aware of the increasing number of artificial intelligence (AI)–based CAs [4] as well as voice-based CAs for health care purposes [53]. We believe this could be an interesting path for future research in this context of personalized patient-CA interaction styles. AI-based CAs could not only interact in a more naturalistic way by utilizing unconstrained written, spoken, or visual input [4], but also further adapt dynamically to personal developments, for example, the level of disease literacy of their human users.

### Suggestions for Future Research

In general, we advise future research to put a stronger focus on the investigation of patients’ personal preferences for specific interaction styles when interacting with CAs based on the long-known importance of this factor in the human patient-human physician context. In detail, we see specific possibilities for future research motivated by the limitations of this study and as an extension of it.

First, we advise future research to expand and test the used systematic approach for inducing two specific interaction styles to more interaction styles. As discussed above in the Strengths and Limitations subsection, we could see variations in preferences when patients could choose from four interaction styles (but only having one written interaction snippet to choose from) and a setting where they could interact for longer with either a deliberative or paternalistic CA. This could provide valuable insights into how many different interaction styles for patient-CA interactions are needed. In addition, we recommend future research to study the development of patients’ preferences over time. It would be highly relevant to determine whether such preferences stay stable or dynamically evolve over time. Second, we suggest the development and evaluation of CAs in other languages besides German and in more diverse geographical settings to investigate the effects of language and regional specificities on patient-CA interaction styles. Third, we recommend focusing on the preferences of patients suffering from different diseases (both acute and chronic). We suggest focusing on differences within as well as between diseases to understand any influencing factors of the medical condition at hand in detail. Fourth, future research could expand our experiment and develop a more extended interaction between patients and interaction style–personalized CAs. This could bear interesting findings to further understand dynamic developments of personal preferences for interaction styles between patients and CAs. Fifth, we believe the development and implementation of AI-based CAs that are able to interact more naturally and adapt dynamically to the patient at hand could yield interesting results in the field of patients’ personal preferences for their interaction with CAs.

### Conclusions

The interaction style between patients and physicians is recognized as a critical parameter for patient satisfaction, treatment adherence, and subsequent treatment outcome and, as such, also plays a paramount role for chronic disease management. So far, CAs as ubiquitous and scalable digital tools have mainly utilized a single interaction style for every patient, thus ignoring the relevance of personalized interaction styles. In this paper, we showed that chronically diseased patients exhibit preferences for different interaction styles when conversing with a digital health CA. Our results provide evidence that patients’ age and personal experiences with the disease inform their preferences for a specific interaction style. Hereby, this work provides insights into the rising trend of personalized CAs in health care. We envisage a future where every chronic patient gets paired with a CA exhibiting the right interaction style at the right moment and dynamically adapting to the needs of the patient, thereby allowing for a satisfying and fulfilling patient-CA interaction that supports the best possible treatment outcomes and disease management.

## Appendix

Multimedia Appendix 1 Online experiment in Study 2 (translated by the authors into English for this paper).

Multimedia Appendix 2 Chronic obstructive pulmonary disease chatbot study flyer (translated by the authors into English for this paper).

Multimedia Appendix 3 Chronic obstructive pulmonary disease chatbot study letter (translated by the authors into English for this paper).

Multimedia Appendix 4 Online experiment in Study 2: Original German measures.

Multimedia Appendix 5 Secondary analyses.

Multimedia Appendix 6 R scripts for Study 1.

Multimedia Appendix 7 R scripts for Study 2.

## Abbreviations

AI: artificial intelligence
BCKQ: Bristol COPD Knowledge Questionnaire
CA: conversational agent
COPD: chronic obstructive pulmonary disease
GOLD: Global Initiative for Chronic Obstructive Lung Disease
ISCED: International Standard Classification of Education
