# Supplementary material for: Personalization of Conversational Agent-Patient Interaction Styles for Chronic Disease Management: Two Consecutive Cross-sectional Questionnaire Studies
Source: J Med Internet Res. 2021 May 26;23(5):e26643. doi: 10.2196/26643 (PMC8190651; doi:10.2196/26643)
Supplement: Multimedia Appendix 1 [file jmir_v23i5e26643_app1.pdf]

Study on the development of a digital health service

Start of Block: 1. Consent

Q1.1 Welcome!
We are very pleased that you are taking the time to participate in this study. This is your way of helping us develop a digital health service!

The following information is intended to inform you about the goals of this study and your participation in it. Please read the information carefully. Participation in the study is only possible via computer.

Study structure
Entry questionnaire (approx. 4 min)
Interaction with a chatbot (approx. 3 min)
Final questionnaire (approx. 10 min)

Risks
No risks are anticipated from participating in this study.

Voluntariness and right to withdraw participation
Participation in this study is voluntary. You have the unrestricted right to terminate your participation in this study at any time without giving reasons and without any disadvantages to you.

Local Ethics Committee
This study does not fall within the scope of the Human Research Act and therefore does not require approval from the Cantonal Ethics Committee to be conducted.

Confidentiality, data protection and possible uses of the data.
Your data will be treated confidentially and will only be published in anonymized and aggregated form at scientific conferences or in scientific journals. Only scientists from research institutions (ETH Zurich, University of St. Gallen) involved in this study will have access to your original data. Your data will be stored on access-protected computers in accordance with the latest data protection regulations and will only be transmitted in encrypted form. No health or other personal data is processed or stored at any time during the interaction with the chatbot that is

part of this study. Only the usage time is collected, stored and analyzed for statistical purposes and for the further development of the service.

Contact
If you have any further questions, please do not hesitate to contact Christoph Gross (christophgross@ethz.ch) or Theresa Schachner (tschachner@ethz.ch) by e-mail.

Do you agree to the terms of participation outlined above?

○ YES, I have carefully read and understood all information and agree to participate voluntarily in this study.

○ NO, I do not wish to participate in this study under these conditions.

End of Block: 1. Consent

Start of Block: 2. Conditions of participation

Q2.1 **This questionnaire is exclusively intended for participants who speak German and are of full age.**

Please confirm by answering the questions below.

Q2.2 **I speak German.**

○ Yes

○ No

Q2.3 **I am of age.**

○ Yes

○ No

Q2.4 **Which operating system do you use?**

- ○ Windows
- ○ Mac
- ○ Linux
- ○ I do not know.

Q2.5 **Which Internet browser do you use?**

- ☐ Chrome
- ☐ Safari
- ☐ Firefox
- ☐ Edge
- ☐ Internet Explorer
- ☐ Opera
- ☐ I do not know.

End of Block: 2. Conditions of participation

Start of Block: 3. Sociodemographic questions

Q3.1 **What is your year of birth?**

▼ < Please Select >

Q3.2 **What is your gender?**

- ○ Male
- ○ Female
- ○ Other: ____

Q3.3 **What is your country of origin?**

- ○ Switzerland
- ○ Germany
- ○ Austria
- ○ Other: ____

Q3.4 **What is your mother tongue?**

- ○ German
- ○ French
- ○ Italian
- ○ Romansh
- ○ Other: ____

Q3.5 **What is your highest level of completed education?**

- ○ No education completed
- ○ Basic vocational training (apprenticeship)
- ○ Professional baccalaureate
- ○ High school baccalaureate
- ○ Higher technical colleges
- ○ Bachelor FH
- ○ Master FH
- ○ Bachelor pedagogical university > Elementary school
- ○ Master pedagogical university > Secondary school
- ○ Bachelor University / Technical university
- ○ Master University / Technical university
- ○ PhD
- ○ Other: ____

End of Block: 3. Sociodemographic questions

Start of Block: 4. Relationship Preference

Q4.1 Two typical " physician-patient relationships" will be introduced below.

Which one do you like better? Please select ONE of the following two options.

◯ **Version 1**

◯ **Version 2**

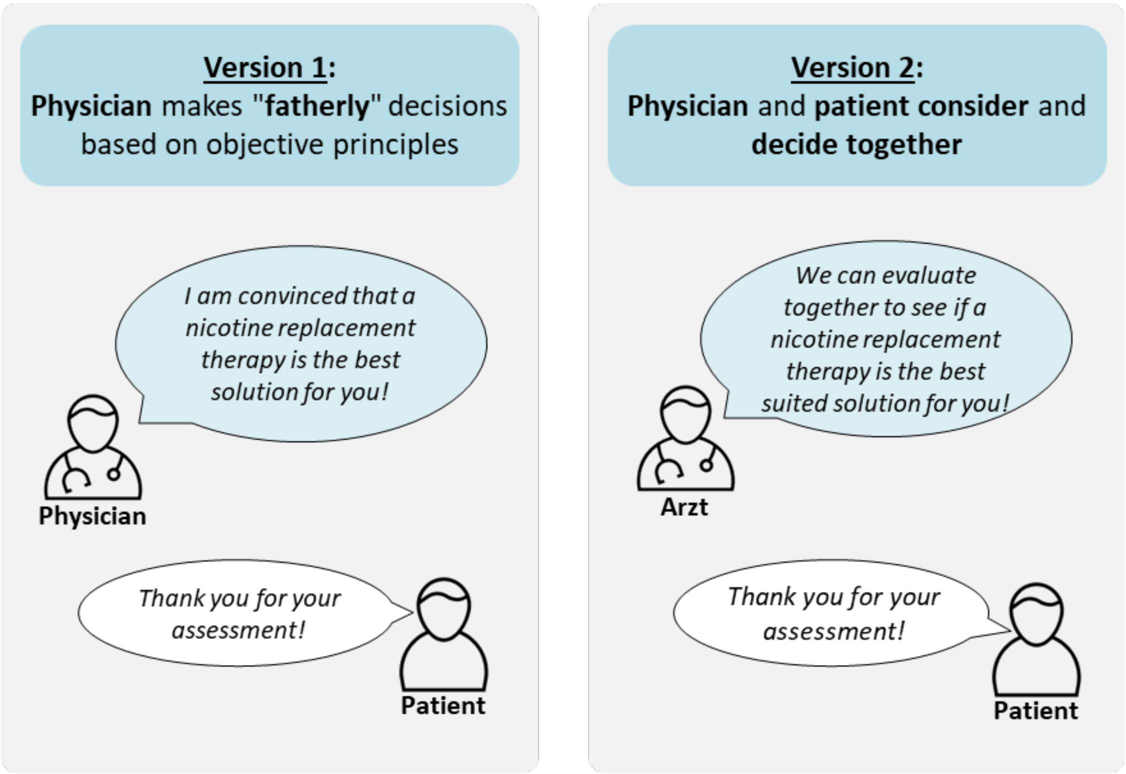

Q4.2 **How do you assess both " physician-patient relationships" versions?**

	Very bad	Bad	Neither good, nor bad	Good	Very good
Version 1: Physician makes "fatherly" decisions based on objective principles	○	○	○	○	○
Version 2: Physician and patient consider and decide together	○	○	○	○	○

End of Block: 4. Relationship Preference

Start of Block: 5. Health-related questions

Q5.1 **Have you been diagnosed with chronic obstructive pulmonary disease / COPD?**

○ Yes

○ No

Page Break

Q5.2 **In which year?**

▼ < Please select >

Q5.3 **Are you currently being treated for your COPD?**

- ◯ Yes
- ◯ No

Q5.4 **In which of the following hospitals?**

- ◯ [Placeholder – Hospital 1]
- ◯ [Placeholder – Hospital 2]
- ◯ [Placeholder – Hospital 3
- ◯ [Placeholder – Hospital 4]
- ◯ Other: ____

Q5.5 **Do you know your "GOLD" classification? If yes, please provide:**

- ◯ GOLD 1
- ◯ GOLD 2
- ◯ GOLD 3
- ◯ GOLD 4
- ◯ I do not know my GOLD classification
- ◯ I do not know what the "GOLD" classification is

Q5.6 **For how many years have you had this GOLD classification?**

- ○ 0-1 years
- ○ 2-5 years
- ○ 6-10 years
- ○ More than 11 years
- ○ I do not have a GOLD classification

Q5.7 **How would you rate your own knowledge of COPD?**
Please select one of the options below.

- ○ Very low
- ○ Low
- ○ Neutral
- ○ High
- ○ Very high

Q5.8 **How confident are you in your ability to explain COPD to another person?**
Please select one of the options below.

- ○ Very low
- ○ Low
- ○ Neutral
- ○ High
- ○ Very high

Page Break

Q5.9 **Have you been diagnosed with any other chronic condition?**

- ○ Yes
- ○ No

Q5.10 **Which chronic disease?**

- ☐ Asthma
- ☐ Dementia
- ☐ Cancer
- ☐ Cardiovascular diseases
- ☐ Chronic lung diseases
- ☐ Epilepsy
- ☐ Multiple sclerosis
- ☐ Rheumatism
- ☐ Diabetes
- ☐ Alcoholism
- ☐ Arteriosclerosis
- ☐ Other: ____

Page Break

Q5.11 **Does someone in your family or close circle of friends suffer from a chronic illness?**

○ Yes

○ No

Q5.12 **Which chronic disease?**

☐ Asthma

☐ Dementia

☐ Cancer

☐ Cardiovascular diseases

☐ Chronic lung diseases

☐ Epilepsy

☐ Multiple sclerosis

☐ Rheumatism

☐ Diabetes

☐ Alcoholism

☐ Arteriosclerosis

☐ Other: ____

Page Break

Q5.13 **Do you currently smoke?**

- ○ Yes
- ○ No, I do not smoke anymore
- ○ No, I have never smoked

Q5.14 **How long have you been smoking?**

- ○ 0–4 years
- ○ 5–10 years
- ○ 11–15 years
- ○ 16–20 years
- ○ More than 20 years

Q5.15 **How many cigarettes do you smoke on average per day?**

- ○ Less than 10
- ○ 11–20
- ○ 21–30
- ○ More than 30

Q5.16 **When did you stop?**

- ○ 0–4 years
- ○ 5–10 years
- ○ 11–15 years
- ○ 16–20 years
- ○ More than 20 years

Q5.17 **On average, how many cigarettes did you smoke per day?**

- ○ Less than 10
- ○ 11–20
- ○ 21–30
- ○ More than 30

Q5.18 **How many years have you smoked in total?**

- ○ Less than 4 years
- ○ 5–10 years
- ○ 11–15 years
- ○ 16–20 years
- ○ More than 20 years

Page Break

End of Block: 5. Health related questions

Start of Block: 6. Chatbot and technology affinity

Q6.1 **Chatbots are computer programs that can converse verbally or in writing in natural language (e.g., German, French). The word chatbot is composed of the English word "to chat" and "bot", short for robot.**

Q6.2 **Have you used a chatbot before?**

- ○ Yes
- ○ No

Q6.3 **Please name any chatbot(s) that you have interacted with before:**

____

Page Break

Q6.4 **How do you think about technical systems?**
Please select one of the options per row.

	completely disagree	largely disagree	slightly disagree	slightly agree	largely agree	completely agree
I like to occupy myself in greater detail with technical systems.	○	○	○	○	○	○
I like testing the functions of new technical systems.	○	○	○	○	○	○
It is enough for me that a technical system works; I don't care how or why.	○	○	○	○	○	○
It is enough for me to know the basic functions of a technical system.	○	○	○	○	○	○

End of Block: 6. Chatbot and technology affinity

Start of Block: 7. Quality-of-Life (COPD Assessment CAT)

Q7.1 **How are you doing with your COPD today? Please complete the COPD Assessment Test™ (CAT)!**

	1	2	3	4	5	6	
I never cough	○	○	○	○	○	○	I cough all the time
I have no phlegm (mucus) in my chest at all	○	○	○	○	○	○	My chest is completely full of phlegm (mucus)
My chest does not feel tight at all	○	○	○	○	○	○	My chest feels very tight
When I walk up a hill or one flight of stairs, I am not breathless	○	○	○	○	○	○	When I walk up a hill or one flight of stairs, I am very breathless
I am not limited doing any activities at home	○	○	○	○	○	○	I am very limited doing activities at home
I am confident leaving my home despite my lung condition	○	○	○	○	○	○	I am not at all confident leaving my home because of my lung condition
I sleep soundly	○	○	○	○	○	○	I don’t sleep soundly because of my lung condition
I have lots of energy	○	○	○	○	○	○	I have no energy at all

End of Block: 7. Quality-of-Life (COPD Assessment CAT)

Start of Block: 8. Chatbot Interaction INFOBOX

Q8.1 **Explanation:**

In the next step, you will have a short interaction with Robo, a chatbot. The interaction works like this:

You will see a screen with speech bubbles appearing. When Robo speaks, you will see a circle with the letters "RB" to the left of the speech bubbles. You will also see one or more white speech bubbles outlined in black. These are your answer choices! Click on the white speech bubble that best corresponds to your personal answer (please only click on one speech bubble!). If there is only one white speech bubble, please click on it. As soon as you have selected an answer, it will be darkened. Now it's Robo's turn again!

At the end of the interaction, we will continue with the questionnaire as before - we will then ask you to answer a few questions about Robo.

<u>Under no circumstances must you click on the browser's "Back" button during the interaction!</u>

End of Block: 8. Chatbot Interaction INFOBOX

Start of Block: 9. Chatbot Interaction Paternalistic

[Placeholder Chatbot Interaction]

End of Block: 9. Chatbot Interaction Paternalistic

Start of Block: 10. Chatbot Interaction Deliberative

[Placeholder Chatbot Interaction]

End of Block: 10. Chatbot Interaction Deliberative

Start of Block: 11. Evaluation of the chatbot interaction

Q11.1 **What is the name of the chatbot you just communicated with?**

- ○ Maral
- ○ Peter
- ○ Robo
- ○ Michel

Page Break

Q11.2 **Which description of the interaction with Robo fits better?**

- ○ Robo makes "paternal" decisions based on objective grounds.
- ○ Robo and I reflect and decide together.

Page Break

Q11.3 **I found the duration of the interaction with the chatbot to be fine.**

Please select one of the choices.

	not at all true	largely not true	rather not true	rather true	largely true	completely true
Please select >>>	○	○	○	○	○	○

Page Break

Q11.4 **I perceived Robo as a "caring father".**

Please select one of the choices.

	not at all true	largely not true	rather not true	rather true	largely true	completely true
Please select >>>	○	○	○	○	○	○

Q11.5 **I perceived Robo as a "friend".**

Please select one of the choices.

	not at all true	largely not true	rather not true	rather true	largely true	completely true
Please select >>>	○	○	○	○	○	○

Page Break

Q11.6 **Could Robo motivate you for the proposed exercise?**

○ Yes

○ No

Q11.7 **I would do the exercise suggested by Robo.**

- not at all true
- largely not true
- rather not true
- rather true
- largely true
- totally true

Page Break

Q11.8 **How would you characterize your relationship with Robo?**

	1	2	3	4	5	
Complete stranger	○	○	○	○	○	Friend

Q11.9 **I think Robo liked me.**

	1	2	3	4	5	
Not at all	○	○	○	○	○	Not at all

Q11.10 **How much do you like Robo?**

	1	2	3	4	5	
Not at all	○	○	○	○	○	Not at all

Page Break

Q11.11 **All in all, I was very pleased with the interaction with Robo.**

Please select one of the choices.

	not at all true	largely not true	rather not true	rather true	largely true	completely true
Please select >>>	○	○	○	○	○	○

Q11.12 **I would like to continue using Robo.**

Please select one of the choices.

	not at all true	largely not true	rather not true	rather true	largely true	completely true
Please select >>>	○	○	○	○	○	○

Q11.13 **How likely is it that you would recommend Robo to a colleague or friend who also has COPD?**

	1	2	3	4	5	6	7	8	9	10
Please select >>>	○	○	○	○	○	○	○	○	○	○

Page Break

Q11.14 **What do you think about Robo, respectively your interaction?**

Below are a series of statements, please decide for each of these statements to what extent it applies to your interaction with Robo.

	rarely	sometimes	frequently	very frequently	always
I think Robo likes me.	○	○	○	○	○
Robo and I respect each other.	○	○	○	○	○
I feel that Robo appreciates me.	○	○	○	○	○
I believe that Robo will stand by me even if I do something, he doesn't approve of.	○	○	○	○	○
Robo and I work together to set therapy goals.	○	○	○	○	○
Robo and I are working toward goals that we agree on.	○	○	○	○	○
Robo and I agree on what is important for me to work on.	○	○	○	○	○
Robo and I are clear about what changes would be good for me.	○	○	○	○	○

Page Break

Q11.15 **What day of the week is today?**

- ○ Monday
- ○ Tuesday
- ○ Wednesday
- ○ Thursday
- ○ Friday
- ○ Saturday
- ○ Sunday

Page Break

Q11.16 **I could imagine that an actual chatbot interaction would look like the one I just saw.**

	not at all true	largely not true	rather not true	rather true	largely true	completely true
Please select >>>	○	○	○	○	○	○

Q11.17 **Do you have any other comments about your interaction with Robo?**

____

____

____

____

____

End of Block: 11. Evaluation of the chatbot interaction

Start of Block: 12. Knowledge query COPD

Q12.1 **Please evaluate the following statements for correctness.**
Please select one of the options per row.

	True	False	I don't know
There is usually a gradual worsening over time with COPD.	○	○	○
More than 80% of COPD cases are caused by cigarette smoking.	○	○	○
COPD is often an inherited disease.	○	○	○
Shortness of breath is caused primarily by bronchoconstriction.	○	○	○
Coughing up mucus is more difficult if you are dehydrated (i.e., have not been drinking enough).	○	○	○
In respiratory infections, the sputum usually changes color (yellow or green).	○	○	○
Exercise/sports should be avoided as this puts a burden on the lungs.	○	○	○
Smoking cessation can slow the progression of lung damage.	○	○	○
Overuse of antibiotics can lead to resistant bacteria (germs).	○	○	○
Indigestion is a common side effect of taking steroid tablets.	○	○	○

End of Block: 12. Knowledge query COPD

Start of Block: 13. Duration

Q13.1 **I found the duration to complete this questionnaire to be fine.**

○ Yes

○ No

End of Block: 13. Duration
