# Supplementary material for: Personalization of Conversational Agent-Patient Interaction Styles for Chronic Disease Management: Two Consecutive Cross-sectional Questionnaire Studies
Source: J Med Internet Res. 2021 May 26;23(5):e26643. doi: 10.2196/26643 (PMC8190651; doi:10.2196/26643)
Supplement: Multimedia Appendix 2 [file jmir_v23i5e26643_app2.pdf]

ETH zürich

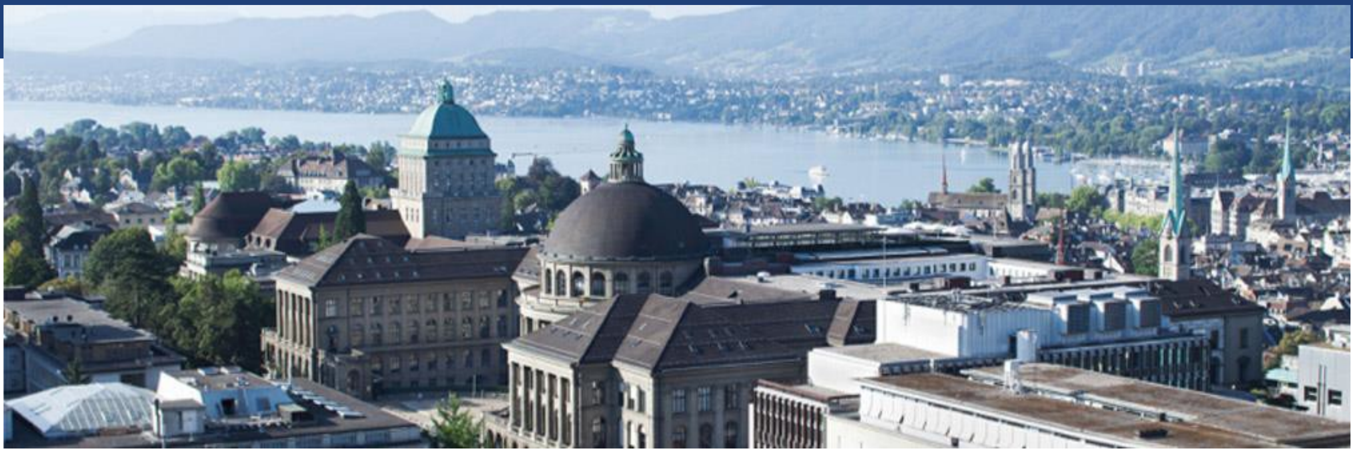

Invitation

Survey on preferences for the design of a chatbot to support COPD patients in their daily lives.

Dear patient,

The Department of Pneumology at the *[Hospital]* is currently conducting a survey with COPD patients in collaboration with the Swiss Federal Institute of Technology Zurich (ETH). The aim of the survey is to collect information about preferences for the design of a chatbot, which is supposed to support patients in their daily routine with their disease. A detailed description of the study can be found on the back of this flyer.

By participating, you do not only help research, but contribute to the development of future chatbot applications that are better tailored to the personal needs of patients.

To participate, you need a computer with access to the Internet. If you use an Apple computer (e.g., MacBook), please open the survey in the Safari browser. If you have a Windows computer, you can use any internet browser. Unfortunately, the survey does not work on cell phones.

[Placeholder – Logo Hospital]

The questionnaire takes about 25 minutes to complete. You do not require any prior knowledge!

To start the survey, please type the following URL into your internet browser:

https://cutt.ly/COPD-Studie-ETH-2020

Thank you very much in advance for your willingness to participate in the study!

Christoph Gross Theresa Schachner

Contact

If you have any questions, please feel free to contact:

Christoph Gross
christophgross@ethz.ch

Theresa Schachner
tschachner@ethz.ch

Study description

Aim of the study

The aim is to better understand differences in your expectations towards a chatbot-based cell phone application (app) that supports you in the day-to-day management of your disease/COPD. More precisely, we are interested in your preferences and behavioral intentions based on the content and features of such an app, which may, for example, assist you in smoking cessation, improving your physical activity, or changing your diet, among other things.

Conditions of participation

The survey is reserved to German-speaking participants of legal age. In addition, participants must have access to a computer with Internet access.

Definition Chatbot

Chatbots are computer programs that can converse (verbally or in writing) with you in natural language (e.g., German, French). The word chatbot is composed of the English word "to chat" and "bot", short version of robot.

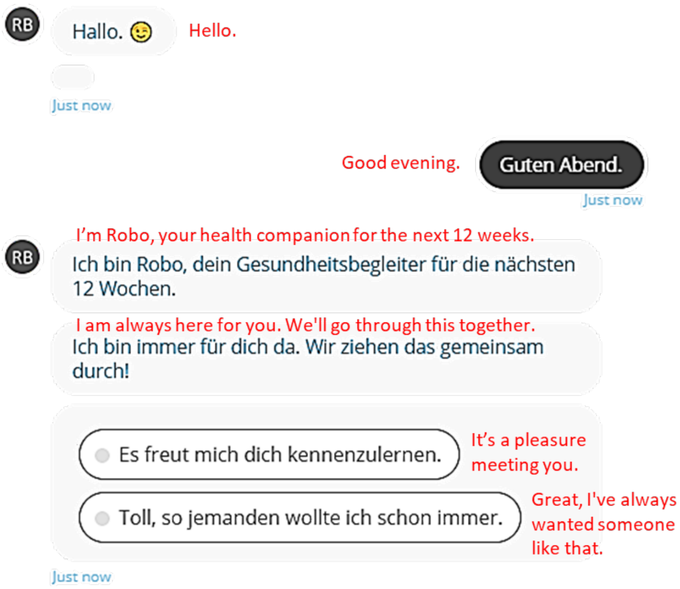

Excerpt of a "chat" with Robo

Study structure/ duration

Opening questionnaire (approx. 10 min)

Interaction with a chatbot (approx. 5 min)

Closing questionnaire (approx. 10 min)

Benefit

With your participation you help research and contribute to further advancement of chatbots.

Risks

No risks are expected from participating in this study.

Voluntariness and right to withdraw participation

The participation in this study is entirely voluntary. You have the unrestricted right to terminate your participation of this study at any time without stating any reasons and with no disadvantages for you.

Local Ethics Committee

This study does not fall within the scope of the Human Research Act and therefore does not require prior approval from the Cantonal Ethics Committee to be conducted.

Confidentiality, data protection and possible uses of the data

Your data will be handled with confidentiality and will only be published in an anonymized and aggregated form at scientific conferences or in scientific journals. Only scientists from research institutions (ETH Zurich, University of St. Gallen) involved in this study will have access to your original data. Your data will be stored on access-protected computers in accordance with current data protection regulations and will only be transmitted in encrypted form. During the interaction with the chatbot, that is part of this study, no health or other personal data will be processed or stored at any time.
