# Supplementary material for: Personalization of Conversational Agent-Patient Interaction Styles for Chronic Disease Management: Two Consecutive Cross-sectional Questionnaire Studies
Source: J Med Internet Res. 2021 May 26;23(5):e26643. doi: 10.2196/26643 (PMC8190651; doi:10.2196/26643)
Supplement: Multimedia Appendix 3 [file jmir_v23i5e26643_app3.pdf]

ETH zürich

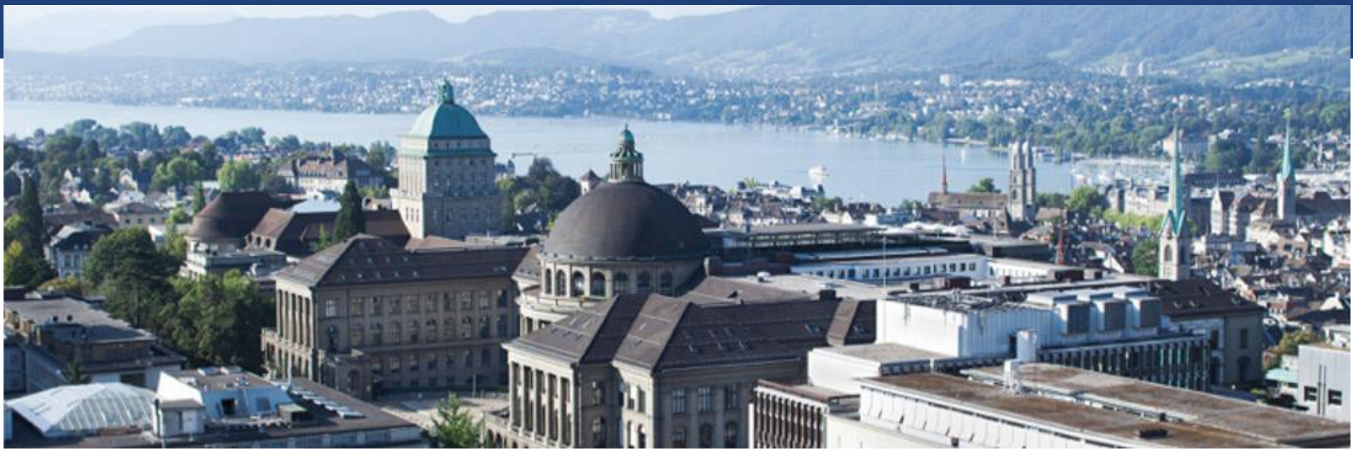

Invitation

Survey on preferences for the design of a chatbot to support COPD patients in their daily lives.

Dear [Placeholder],

We are a small team of researchers at ETH Zurich working on chatbots and their potential use with chronically ill patients. Chatbots are "computer programs that are able to communicate with users in a human-like manner ". We are currently conducting a chatbot study in collaboration with the Pneumology Department of the *[hospital],* in particular with the team around PD Dr. med. *[placeholder].* The survey is conducted online and entirely anonymous. It will take about 20 minutes to complete. The aim of the study is to investigate the preferences of COPD patients regarding their relationship with a "digital physician" (in this case a chatbot) [based on the physician-patient models of Emanuel, E. J., & Emanuel, L. L. (1992). Physician-patient relationship. Jama, 267(16), 16th].

The Cantonal Ethics Committee of Zurich has already given us confirmation that the study does not require ethics approval, as it does not fall within the scope of the Human Research Act.

In order to be able to reach a sufficiently large collective of participants for our survey, we would like to ask you whether you could provide us with contact details of your patients, or send the link to the survey directly to them? In case of any questions, please do not hesitate to contact us. We would of course also send you the final questionnaire in advance for your information. Please find attached an information flyer, which can as well be distributed to your patients.

We are looking forward to your feedback!

Kind regards,

Christoph Gross Theresa Schachner

Contact

If you have any questions, please feel free to contact:

Christoph Gross
christophgross@ethz.ch

Theresa Schachner
tschachner@ethz.ch
