# Supplementary material for: Personalization of Conversational Agent-Patient Interaction Styles for Chronic Disease Management: Two Consecutive Cross-sectional Questionnaire Studies
Source: J Med Internet Res. 2021 May 26;23(5):e26643. doi: 10.2196/26643 (PMC8190651; doi:10.2196/26643)
Supplement: Multimedia Appendix 4 [file jmir_v23i5e26643_app4.pdf]

ETH zürich D MTEC

COPD Umfrage

## 1. Consent

*F1.1.*

### Herzlich Willkommen!

Wir freuen uns sehr, dass Sie sich Zeit nehmen, um an dieser Studie teilzunehmen. So unterstützen Sie uns bei der Entwicklung eines digitalen Gesundheitsservices!

Die folgenden Informationen sind dazu da, Sie über die Ziele dieser Studie und Ihre Beteiligung darin zu informieren. Bitte lesen Sie diese Informationen sorgfältig durch. Die Teilnahme an der Studie ist nur via Computer möglich.

Studienstruktur
Eingangsbefragung (ca. 4 min)
Interaktion mit einem Chatbot (ca. 3 min)
Abschlussfragebogen (ca. 10 min)

Risiken
Es werden keine Risiken durch die Teilnahme an dieser Studie erwartet.

Freiwilligkeit und Recht auf Widerruf der Teilnahme
Die Teilnahme an dieser Studie ist freiwillig. Sie haben das uneingeschränkte Recht, Ihre Teilnahme an dieser Studie zu jedem beliebigen Zeitpunkt ohne Angabe von Gründen zu beenden, ohne dass dadurch Nachteile für Sie entstehen.

Lokale Ethikkommission
Diese Studie fällt nicht in den Geltungsbereich des Humanforschungsgesetzes und bedarf deshalb für die Durchführung keiner Bewilligung der Kantonalen Ethikkommission.

Vertraulichkeit, Datenschutz und Verwendungsmöglichkeiten der Daten
Ihre Daten werden vertraulich behandelt und nur in anonymisierter und aggregierter Form auf wissenschaftlichen Konferenzen oder in wissenschaftlichen Zeitschriften publiziert. Ausschliesslich Wissenschaftler von Forschungseinrichtungen (ETH Zürich, Universität St. Gallen), die an dieser Studie beteiligt sind, können Ihre

Originaldaten einsehen. Ihre Daten werden gemäss den aktuellsten Datenschutzbestimmungen auf zugriffsgeschützten Computern gespeichert und nur verschlüsselt übermittelt.
Innerhalb der Interaktion mit dem Chatbot, die Teil dieser Studie ist, werden zu keinem Zeitpunkt Gesundheits- oder andere personenbezogene Daten bearbeitet oder gespeichert. Es wird nur die Nutzungsdauer zu statistischen Zwecken und zur Weiterentwicklung des Services erhoben, gespeichert und analysiert.

Kontakt
Bei weiteren Fragen können Sie sich gerne jederzeit per E-Mail an Christoph Gross (christophgross@ethz.ch) oder Theresa Schachner (tschachner@ethz.ch) wenden.

**Sind Sie mit den hier beschriebenen Teilnahmebedingungen einverstanden?**

- ○ JA, ich habe alle Informationen sorgfältig gelesen und verstanden und erkläre mich bereit, freiwillig an dieser Studie teilzunehmen.
- ○ NEIN, unter diesen Bedingungen möchte ich nicht an dieser Studie teilnehmen.

## 2. Teilnahmebedingungen

*F2.1.* **Dieser Fragebogen richtet sich ausschliesslich an Teilnehmende, welche Deutsch sprechen und volljährig sind.**

**Bitte bestätigen Sie dies mit der Beantwortung der nachstehenden Fragen.**

*F2.2.* **Ich spreche Deutsch.**

- ○ Ja
- ○ Nein

*F2.3.* **Ich bin volljährig.**

- ○ Ja
- ○ Nein

*F2.4.* **Welches Betriebssystem nutzen Sie?**

- ○ Windows
- ○

Mac

○ Linux

○ Weiss ich nicht.

F2.5. **Welchen Internetbrowser nutzen Sie?**

☐ Chrome
☐ Safari
☐ Firefox
☐ Edge
☐ Internet Explorer
☐ Opera
☐ Weiss ich nicht.

## 3. Soziodemographische Fragen

F3.1. **Welches ist Ihr Geburtsjahr?**

F3.2. **Was ist Ihr Geschlecht?**

○ Männlich

○ Weiblich

○ Anderes:

F3.3. **Welches ist Ihr Herkunftsland?**

○ Schweiz

○ Deutschland

○ Österreich

○ Anderes:

F3.4. **Was ist Ihre Muttersprache?**

○ Deutsch
○ Französisch
○ Rätoromanisch
○ Andere:

- ◯ Italienisch

### *F3.5.* Was ist Ihre höchste abgeschlossene Ausbildung?

- ◯ Keine Ausbildung abgeschlossen
- ◯ Berufliche Grundbildung (Lehre)
- ◯ Berufsmaturität / Fachautorität
- ◯ Gymnasiale Maturität
- ◯ Höhere Fachschulen (HF)
- ◯ Bachelor FH
- ◯ Master FH
- ◯ Bachelor PH > Grundschule
- ◯ Master PH > Sekundarschule
- ◯ Bachelor Universität / ETH
- ◯ Master Universität / ETH
- ◯ Doktorat (PhD)
- ◯ Andere: ______

## 4. Beziehungspräferenz

### *F4.1.* Nachfolgend werden Ihnen zwei verschiedene typische „Arzt-Patienten Beziehungen“ vorgestellt.

**Welches Modell gefällt Ihnen besser? Bitte wählen Sie EINE der folgenden zwei Möglichkeiten aus.**

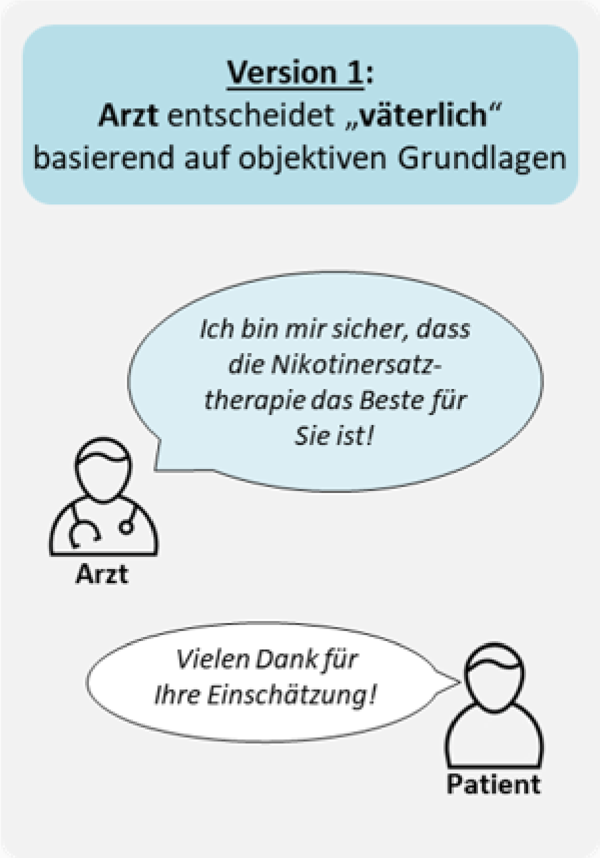


**Version 1**
◯

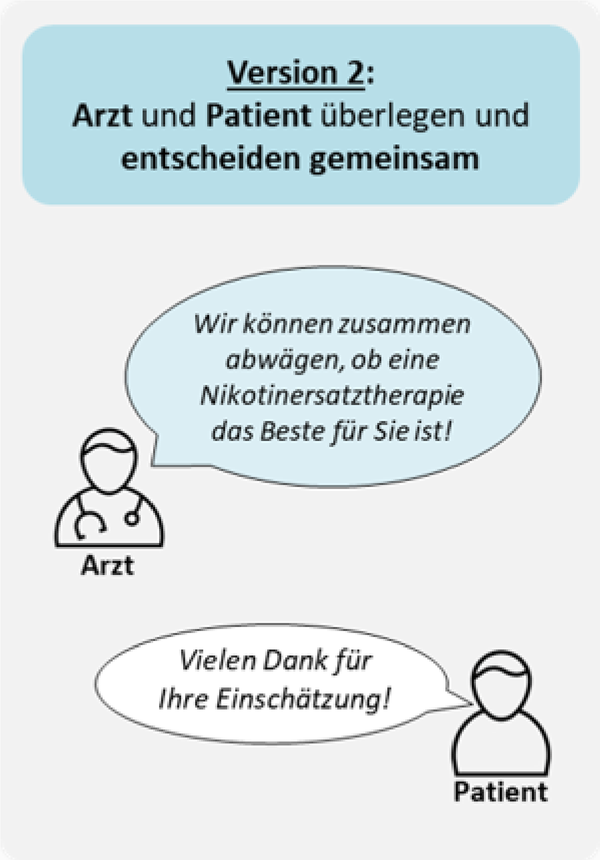


**Version 2**
◯

*F4.2.* **Wie beurteilen Sie beide „Arzt-Patienten Beziehungen“ Varianten?**

| | sehr schlecht | schlecht | weder gut, noch schlecht | gut | sehr gut |
|---|---|---|---|---|---|
| Version 1: **Arzt** entscheidet „**väterlich**“ basierend auf objektiven Grundlagen | ○ | ○ | ○ | ○ | ○ |
| Version 2: **Arzt** und **Patient** überlegen und **entscheiden gemeinsam** | ○ | ○ | ○ | ○ | ○ |

## 5. Gesundheitsbezogene Fragen

*F5.1.* **Wurde bei Ihnen eine chronisch obstruktive Lungenerkrankung bzw. COPD festgestellt?**

○ Ja

○ Nein

*F5.2.* **In welchem Jahr?**

*F5.3.* **Sind Sie derzeit in Behandlung wegen Ihrer COPD?**

○ Ja

○ Nein

*F5.4.* **In welchem der folgenden Krankenhäuser?**

○ [Platzhalter - Krankenhaus 1]

○ [Platzhalter - Krankenhaus 2]

○ [Platzhalter - Krankenhaus 3]

○ [Platzhalter - Krankenhaus 4]

○ Anderes:

*F5.5.* **Kennen Sie Ihre „GOLD“ Einstufung? Wenn ja, bitte geben Sie diese an:**

- ○ GOLD I
- ○ GOLD II
- ○ GOLD III
- ○ GOLD IV
- ○ Ich kenne meine GOLD Einstufung nicht
- ○ Ich weiss nicht, was die „GOLD“ Einstufung ist

*F5.6.* **Seit wie vielen Jahren haben Sie diese GOLD Einstufung?**

- ○ 0-1 Jahre
- ○ 2-5 Jahre
- ○ 6-10 Jahre
- ○ mehr als 11 Jahre
- ○ Ich habe keine GOLD Einstufung

*F5.7.* **Wie würden Sie Ihre Kenntnisse über COPD selbst einschätzen?**
*Bitte wählen Sie eine der Auswahlmöglichkeiten aus.*

| sehr niedrig | nierdrig | neutral | hoch | sehr hoch |
|---|---|---|---|---|
| ○ | ○ | ○ | ○ | ○ |

*F5.8.* **Wie sicher fühlen Sie sich, einer anderen Person erklären zu können, was COPD ist?**
*Bitte wählen Sie eine der Auswahlmöglichkeiten aus.*

| sehr niedrig | nierdrig | neutral | hoch | sehr hoch |
|---|---|---|---|---|
| ○ | ○ | ○ | ○ | ○ |

*F5.9.* **Wurden bei Ihnen andere chronische Erkrankungen festgestellt?**

- ○ Ja
- ○ Nein

*F5.10.* **Welche chronische Erkrankung?**

- ☐ Asthma
- ☐ Demenz
- ☐ Krebs
- ☐ Herz-Kreislauf-Erkrankungen
- ☐ Chronische Lungenerkrankungen
- ☐ Epilepsie
- ☐ Multiple Sklerose
- ☐ Rheuma
- ☐ Diabetes
- ☐ Alkoholismus
- ☐ Arteriosklerose
- ☐ Andere: ____

*F5.11.* **Leidet jemand in Ihrer Familie oder Ihrem engem Freundeskreis an einer chronischen Erkrankung?**

- ○ Ja
- ○ Nein

*F5.12.* **Welche chronische Erkrankung?**

- ☐ Asthma
- ☐ Demenz
- ☐ Krebs
- ☐ Herz-Kreislauf-Erkrankungen
- ☐ Chronische Lungenerkrankungen
- ☐ Epilepsie
- ☐ Multiple Sklerose
- ☐ Rheuma
- ☐ Diabetes
- ☐ Alkoholismus
- ☐ Arteriosklerose
- ☐ Andere: ____

*F5.13.* **Rauchen Sie aktuell?**

- ○ Ja
- ○ Nein, ich rauche ***nicht mehr***
- ○ Nein, ich habe ***nie*** geraucht

*F5.14.* **Wie lange rauchen Sie schon?**

- ○ 0–4 Jahre
- ○ 5–10 Jahre
- ○ 11–15 Jahre
- ○ 16–20 Jahre
- ○ mehr als 20 Jahre

*F5.15.* **Wie viele Zigaretten rauchen Sie im Durchschnitt pro Tag?**

- ○ weniger als 10
- ○ 11–20
- ○ 21–30
- ○ mehr als 30

*F5.16.* **Wann haben Sie aufgehört? Vor...**

- ○ 0–4 Jahren
- ○ 5–10 Jahren
- ○ 11–15 Jahren
- ○ 16–20 Jahren
- ○ mehr als 20 Jahren

*F5.17.* **Wie viele Zigaretten haben Sie im Durchschnitt pro Tag geraucht?**

- ○ weniger als 10
- ○ 11–20
- ○ 21–30
- ○ mehr als 30

*F5.18.* **Wie viele Jahre haben Sie insgesamt geraucht?**

- ○ weniger als 4 Jahre
- ○ 5–10 Jahre
- ○ 11–15 Jahre
- ○ 16–20 Jahre
- ○ mehr als 20 Jahre

## 6. Chatbot und Technologieaffinität

*F6.1.*
**Chatbots sind eine Art Computerprogramm, mit dem man sich in natürlicher Sprache (z.B.: Deutsch, Französisch) schriftlich oder**

**mündlich unterhalten kann. Das Wort Chatbot setzt sich zusammen aus dem englischen Wort “to chat” (sich unterhalten) und “bot”, kurz für Roboter.**

*F6.2.* **Haben Sie selbst bereits einen Chatbot genutzt?**

- ○ Ja
- ○ Nein

*F6.3.* **Bitte nennen Sie einen oder mehrere Chatbots, mit denen Sie bereits einmal gechattet haben:**

*F6.4.* **Wie denken Sie über technische Systeme?**
*Bitte wählen Sie jeweils eine der Auswahlmöglichkeiten pro Zeile aus.*

| | stimmt gar nicht | stimmt weitgehend nicht | stimmt eher nicht | stimmt eher | stimmt weitgehend | stimmt völlig |
|---|---|---|---|---|---|---|
| Ich beschäftige mich gern genauer mit technischen Systemen. | ○ | ○ | ○ | ○ | ○ | ○ |
| Ich probiere gern die Funktionen neuer technischer Systeme aus. | ○ | ○ | ○ | ○ | ○ | ○ |
| Es genügt mir, dass ein technisches System funktioniert, mir ist es egal, wie oder warum. | ○ | ○ | ○ | ○ | ○ | ○ |
| Es genügt mir, die Grundfunktionen eines technischen Systems zu kennen. | ○ | ○ | ○ | ○ | ○ | ○ |

## 7. Lebensqualität (COPD Assessment CAT)

*F7.1.* **Wie geht es Ihnen heute mit Ihrer COPD? Füllen Sie den COPD Assessment Test™ (CAT) aus!**

| | 1 | 2 | 3 | 4 | 5 | 6 | |
|---|---|---|---|---|---|---|---|
| Ich huste nie. | ○ | ○ | ○ | ○ | ○ | ○ | Ich huste ständig. |

| | | |
|---|---|---|
| Ich bin überhaupt nicht verschleimt. | ○ ○ ○ ○ ○ ○ | Ich bin völlig verschleimt. |
| Ich spüre keinerlei Engegefühl in der Brust. | ○ ○ ○ ○ ○ ○ | Ich spüre ein sehr starkes Engegefühl in der Brust. |
| Wenn ich bergauf oder eine Treppe hinaufgehe, komme ich nicht ausser Atem. | ○ ○ ○ ○ ○ ○ | Wenn ich bergauf oder eine Treppe hinaufgehe, komme ich sehr ausser Atem. |
| Ich bin bei meinen häuslichen Aktivitäten nicht eingeschränkt. | ○ ○ ○ ○ ○ ○ | Ich bin bei meinen häuslichen Aktivitäten sehr stark eingeschränkt. |
| Ich habe keine Bedenken, trotz meiner Lungenerkrankung, das Haus zu verlassen. | ○ ○ ○ ○ ○ ○ | Ich habe wegen meiner Lungenerkrankung grosse Bedenken, das Haus zu verlassen. |
| Ich schlafe tief und fest. | ○ ○ ○ ○ ○ ○ | Wegen meiner Lungenerkrankung schlafe ich nicht tief und fest. |
| Ich bin voller Energie. | ○ ○ ○ ○ ○ ○ | Ich habe überhaupt keine Energie. |

## 8. Chatbot Interaktion INFOBOX

*F8.1.*
**Erklärung:**

Im nächsten Schritt werden Sie eine kurze Interaktion mit Robo, einem Chatbot, führen.

Die Interaktion funktioniert so:

Sie sehen einen Bildschirm, auf dem Sprechblasen auftauchen (siehe **Bild 1 & 2**). Wenn Robo spricht, sehen Sie links neben seinen Sprechblasen einen Kreis mit den Buchstaben «RB». Sie sehen auch eine oder mehrere weisse Sprechblasen, die schwarz umrandet sind. Das sind Ihre Antwortmöglichkeiten! Klicken Sie auf die weisse Sprechblase, die Ihrer persönlichen Antwort am besten entspricht (bitte nur auf eine Sprechblase klicken!). **Bild 1** zeigt diesen Vorgang. Wenn es nur eine weisse Sprechblase gibt, klicken Sie bitte auf diese (siehe **Bild 2**). Sobald Sie eine Antwort ausgewählt haben, wird diese dunkel hinterlegt (siehe **Bild 2**). Nun ist Robo wieder an der Reihe!

Am Ende der Interaktion geht es ganz normal mit dem Fragebogen weiter - wir werden Sie bitten ein paar Fragen zu Robo zu beantworten.

Bitte klicken Sie während der Interaktion auf keinen Fall auf den "Zurück"-Button des

Browsers!

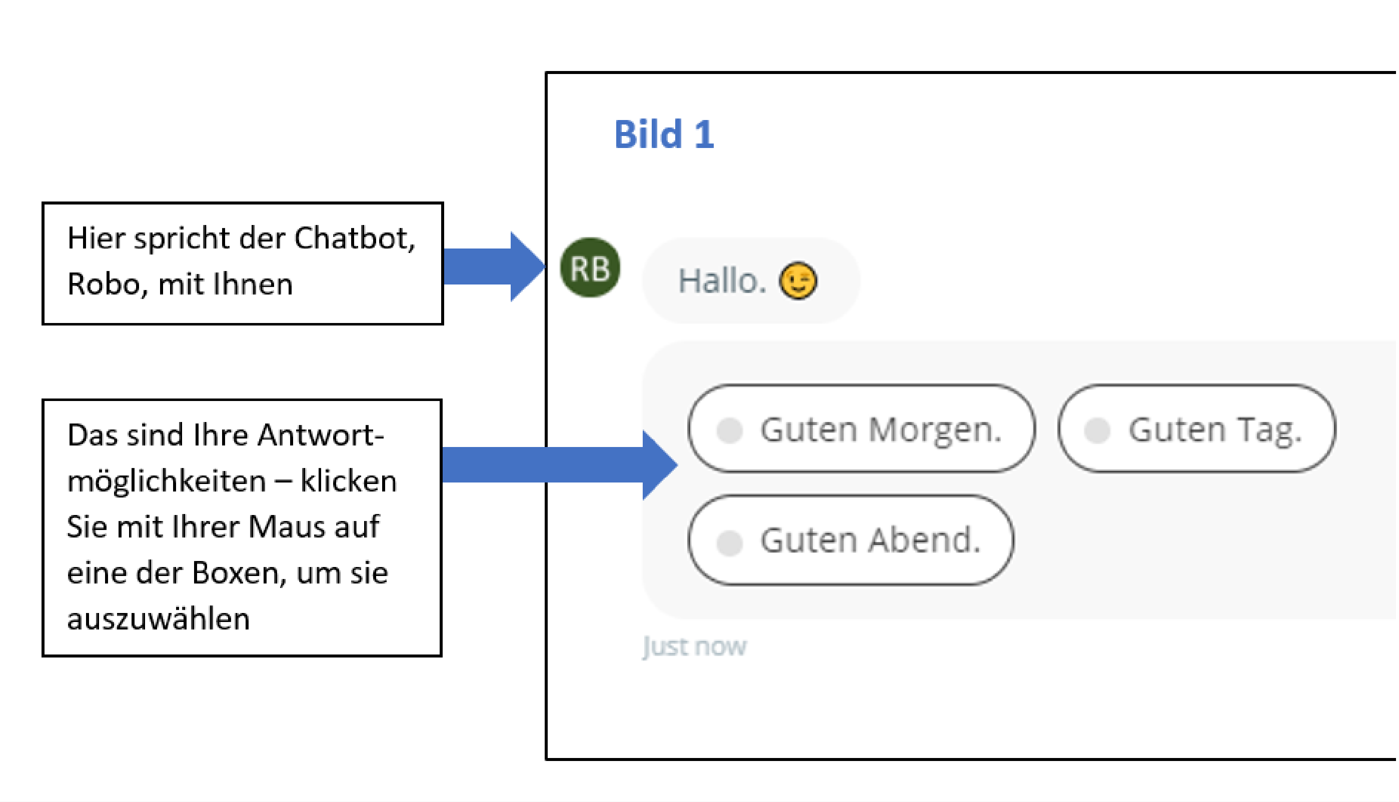
Bild 1
Hier spricht der Chatbot, Robo, mit Ihnen
Das sind Ihre Antwort-möglichkeiten – klicken Sie mit Ihrer Maus auf eine der Boxen, um sie auszuwählen
RB
Hallo.
Guten Morgen.
Guten Tag.
Guten Abend.
Just now

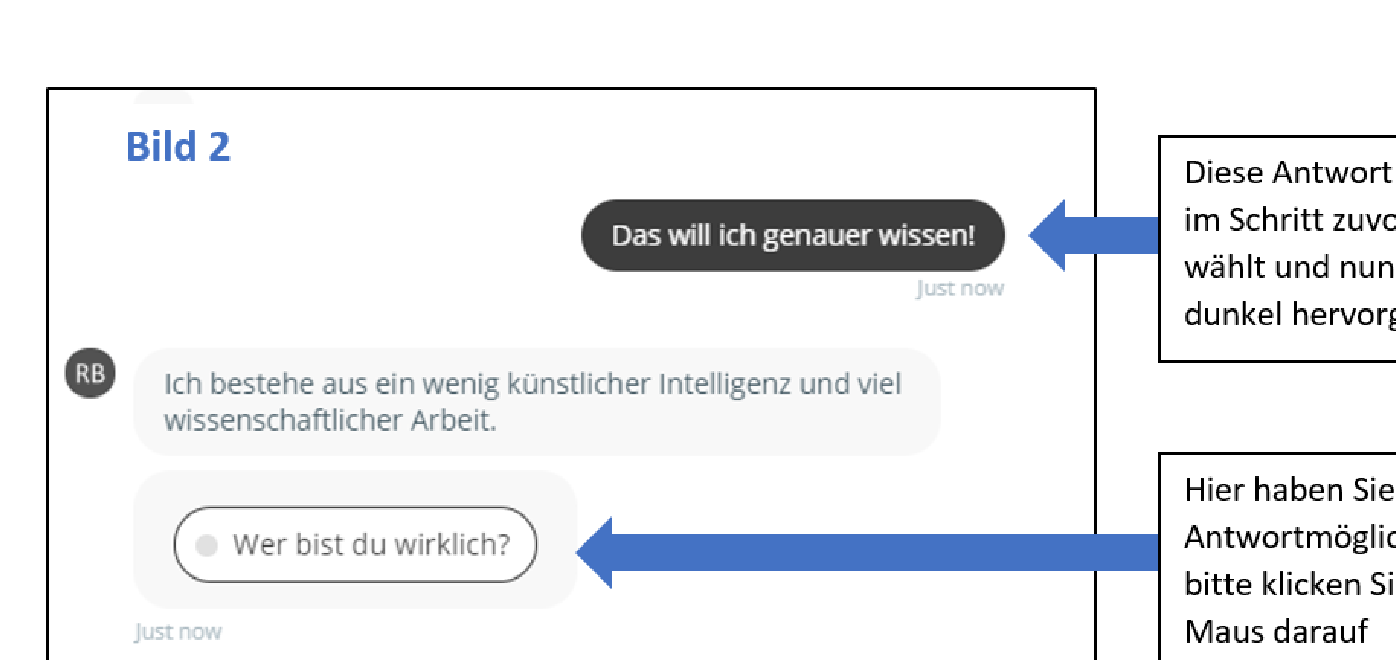
Bild 2
Das will ich genauer wissen!
Just now
RB
Ich bestehe aus ein wenig künstlicher Intelligenz und viel wissenschaftlicher Arbeit.
Wer bist du wirklich?
Just now
Diese Antwort im Schritt zuvo wählt und nun dunkel hervorg
Hier haben Sie Antwortmöglic bitte klicken Si Maus darauf

## 9. Chatbot Interaktion Paternalistisch

**These page timer metrics will not be displayed to the recipient.**
First Click: *0 seconds*
Last Click: *0 seconds*
Page Submit: *0 seconds*
Click Count: *0 clicks*

*F9.2.*

Hallo. 😉

Guten Morgen. Guten Tag.

Guten Abend.

Just now

Chat by Collect.chat

## 10. Chatbot Interaktion Deliberativ

**These page timer metrics will not be displayed to the recipient.**
First Click: *0 seconds*
Last Click: *0 seconds*
Page Submit: *0 seconds*
Click Count: *0 clicks*

*F10.2.*

Hallo. 😉

Guten Morgen.

Guten Tag.

Guten Abend.

Just now

Chat by Collect.chat

## 11. Bewertung der Chatbot Interaktion

*F11.1.* **Wie heisst der Chatbot mit dem Sie gerade kommuniziert haben?**

- ○ Maral
- ○ Peter
- ○ Robo
- ○ Michel

*F11.2.* **Welche Beschreibung der Interaktion mit Robo passt besser?**

○ Robo entscheidet „**väterlich**“ basierend auf **objektiven Grundlagen**.

○ **Robo** und **ich** überlegen und **entscheiden gemeinsam.**

*F11.3.* **Die Dauer der Interaktion mit dem Chatbot fand ich in Ordnung.**
Bitte wählen Sie eine der Auswahlmöglichkeiten aus.

| | stimmt gar nicht | stimmt weitgehend nicht | stimmt eher nicht | stimmt eher | stimmt weitgehend | stimmt völlig |
|---|---|---|---|---|---|---|
| Bitte auswählen >>> | ○ | ○ | ○ | ○ | ○ | ○ |

*F11.4.* **Ich habe Robo als "fürsorglichen Vater“ wahrgenommen.**
*Bitte wählen Sie eine der Auswahlmöglichkeiten aus.*

| | stimmt gar nicht | stimmt weitgehend nicht | stimmt eher nicht | stimmt eher | stimmt weitgehend | stimmt völlig |
|---|---|---|---|---|---|---|
| Bitte auswählen >>> | ○ | ○ | ○ | ○ | ○ | ○ |

*F11.5.* **Ich habe Robo als "Freund“ wahrgenommen.**
*Bitte wählen Sie eine der Auswahlmöglichkeiten aus.*

| | stimmt gar nicht | stimmt weitgehend nicht | stimmt eher nicht | stimmt eher | stimmt weitgehend | stimmt völlig |
|---|---|---|---|---|---|---|
| Bitte auswählen >>> | ○ | ○ | ○ | ○ | ○ | ○ |

*F11.6.* **Konnte Robo Sie für die vorgeschlagene Übung motivieren?**

○ Ja

○ Nein

*F11.7.* **Ich würde die von Robo vorgeschlagene Übung machen.**

| stimmt gar nicht | stimmt weitgehend nicht | stimmt eher nicht | stimmt eher | stimmt weitgehend | stimmt völlig |
|---|---|---|---|---|---|
| ○ | ○ | ○ | ○ | ○ | ○ |

*F11.8.* **Wie würden Sie Ihre Beziehung zu Robo charakterisieren?**

Völlig fremder Mensch ○ ○ ○ ○ ○ Freund

*F11.9.* **Ich glaube Robo mochte mich.**

Überhaupt nicht ○ ○ ○ ○ ○ Sehr gern

*F11.10.* **Wie sehr mögen Sie Robo?**

Überhaupt nicht ○ ○ ○ ○ ○ Sehr gern

*F11.11.* **Alles in allem war ich mit der Interaktion mit Robo sehr zufrieden.**
*Bitte wählen Sie eine der Auswahlmöglichkeiten aus.*

| | stimmt gar nicht | stimmt weitgehend nicht | stimmt eher nicht | stimmt eher | stimmt weitgehend | stimmt völlig |
|---|---|---|---|---|---|---|
| Bitte auswählen >>> | ○ | ○ | ○ | ○ | ○ | ○ |

*F11.12.* **Ich würde Robo gerne weiter verwenden.**
*Bitte wählen Sie eine der Auswahlmöglichkeiten aus.*

| | stimmt gar nicht | stimmt weitgehend nicht | stimmt eher nicht | stimmt eher | stimmt weitgehend | stimmt völlig |
|---|---|---|---|---|---|---|
| Bitte auswählen >>> | ○ | ○ | ○ | ○ | ○ | ○ |

*F11.13.* **Wie wahrscheinlich ist es, dass Sie Robo einem Kollegen oder Freund, der auch an COPD leidet, weiterempfehlen würden?**

sehr unwahrscheinlich (0) sehr wahrscheinlich (10)

| | 1 | 2 | 3 | 4 | 5 | 6 | 7 | 8 | 9 | 10 |
|---|---|---|---|---|---|---|---|---|---|---|
| Bitte auswählen >>> | ○ | ○ | ○ | ○ | ○ | ○ | ○ | ○ | ○ | ○ |

*F11.14.* **Was denken Sie über Robo, bzw. Ihre gemeinsame Interaktion?**

Im folgenden finden Sie eine Reihe von Aussagen, bitte entscheiden Sie bei jeder dieser Aussage, inwieweit sie auf Ihre Interaktion mit Robo zutrifft.

| | selten | manchmal | öfter | sehr oft | immer |
|---|---|---|---|---|---|

| | selten | manchmal | öfter | sehr oft | immer |
|---|---|---|---|---|---|
| Ich glaube, Robo mag mich. | ○ | ○ | ○ | ○ | ○ |
| Robo und ich respektieren einander. | ○ | ○ | ○ | ○ | ○ |
| Ich habe das Gefühl, dass Robo mich schätzt. | ○ | ○ | ○ | ○ | ○ |
| Ich glaube, dass Robo auch dann zu mir steht, wenn ich etwas tue, was er/sie nicht gutheisst. | ○ | ○ | ○ | ○ | ○ |
| Robo und ich arbeiten gemeinsam daran, Therapieziele zu setzen. | ○ | ○ | ○ | ○ | ○ |
| Robo und ich arbeiten auf Ziele hin, über die wir uns einig sind. | ○ | ○ | ○ | ○ | ○ |
| Robo und ich stimmen überein, woran es für mich wichtig ist zu arbeiten. | ○ | ○ | ○ | ○ | ○ |
| Robo und ich sind uns im Klaren darüber, welche Veränderungen gut für mich wären. | ○ | ○ | ○ | ○ | ○ |

### *F11.15.* **Welcher Wochentag ist heute?**

- ○ Montag
- ○ Dienstag
- ○ Mittwoch
- ○ Donnerstag
- ○ Freitag
- ○ Samstag
- ○ Sonntag

### *F11.16.* **Ich könnte mir vorstellen, dass eine echte Chatbot-Interaktion so aussieht wie die, die ich gerade gesehen habe.**

| | stimmt gar nicht | stimmt weitgehend nicht | stimmt eher nicht | stimmt eher | stimmt weitgehend | stimmt völlig |
|---|---|---|---|---|---|---|
| Bitte auswählen >>> | ○ | ○ | ○ | ○ | ○ | ○ |

*F11.17.* **Haben Sie sonst noch Anmerkungen zu Ihrer Interaktion mit Robo?**

## 12. Wissensabfrage COPD

*F12.1.* **Bitte bewerten Sie folgende Aussagen auf Richtigkeit.**
Bitte wählen Sie jeweils eine der Auswahlmöglichkeiten pro Zeile aus.

| | Richtig | Fa |
|---|---|---|
| Bei COPD kommt es im Laufe der Zeit in der Regel zu einer allmählichen Verschlechterung. | ○ | |
| Mehr als 80% der COPD-Fälle werden durch Zigarettenrauchen verursacht. | ○ | |
| COPD ist häufig eine vererbte Erkrankung. | ○ | |
| Luftnot wird vor allem durch eine Verengung der Bronchien verursacht. | ○ | |
| Das Abhusten von Schleim ist schwieriger, wenn Sie dehydriert sind (d.h. nicht genug getrunken haben). | ○ | |
| Bei Atemwegsinfekten verfärbt sich der Auswurf normalerweise (gelb oder grün). | ○ | |
| Bewegung/Sport sollte vermieden werden, da hiermit die Lunge belastet wird. | ○ | |
| Durch das Einstellen des Rauchens kann das Fortschreiten von Lungenschäden verlangsamt werden. | ○ | |
| Übermäßiger Einsatz von Antibiotika kann zu resistenten Bakterien (Keimen) führen. | ○ | |
| Verdauungsstörungen sind eine häufige Nebenwirkung bei der Einnahme von Steroidtabletten. | ○ | |

## 13. Dauer

*F13.1.* **Die Dauer zur Beantwortung dieses Fragebogens fand ich in Ordnung.**

○ Ja

○ Nein

Survey Powered By Qualtrics
