# Supplementary material for: Personalization of Conversational Agent-Patient Interaction Styles for Chronic Disease Management: Two Consecutive Cross-sectional Questionnaire Studies
Source: J Med Internet Res. 2021 May 26;23(5):e26643. doi: 10.2196/26643 (PMC8190651; doi:10.2196/26643)
Supplement: Multimedia Appendix 5 [file jmir_v23i5e26643_app5.docx]

**Table S1.** This table describes the regression of CA type, demographics, and COPD related characteristics on participants' satisfaction (Item 1) with the CA interaction.

|  | Model 1 | Model 2 | Model 3 |
| --- | --- | --- | --- |
|  |  |  |  |
| Intercept | -0.008 | -0.068 | -0.150 |
|  | (-0.262, 0.246) | (-0.397, 0.262) | (-0.646, 0.345) |
| CA Type | 0.016 | 0.064 | 0.160 |
|  | (-0.343, 0.376) | (-0.424, 0.553) | (-0.528, 0.848) |
| Gender |  | -0.002 | 0.483 |
|  |  | (-0.608, 0.604) | (-0.427, 1.392) |
| Age |  | 0.036 | 0.366 |
|  |  | (-0.255, 0.327) | (-0.125, 0.857) |
| Education |  | -0.064 | 0.148 |
|  |  | (-0.346, 0.218) | (-0.269, 0.564) |
| Gender*CA Type |  | -0.001 | -0.779 |
|  |  | (-0.824, 0.822) | (-1.934, 0.375) |
| Age*CA Type |  | -0.068 | -0.602 |
|  |  | (-0.455, 0.319) | (-1.201, -0.003) |
| Education*CA Type |  | 0.036 | -0.100 |
|  |  | (-0.358, 0.429) | (-0.628, 0.429) |
| GOLD |  |  | 0.382 |
|  |  |  | (-0.010, 0.774) |
| COPD Literacy |  |  | 0.318 |
|  |  |  | (-0.210, 0.846) |
| Experience |  |  | -0.325 |
|  |  |  | (-0.741, 0.091) |
| GOLD*CA Type |  |  | -0.162 |
|  |  |  | (-0.711, 0.387) |
| COPD Literacy*CA Type |  |  | 0.009 |
|  |  |  | (-0.619, 0.637) |
| Experience* CA Type |  |  | 0.481 |
|  |  |  | (-0.090, 1.052) |
|  |  |  |  |
| Observations | 120 | 113 | 67 |
| R^2^ | 0.0001 | 0.005 | 0.220 |

*Note.* Wording Item 1: “How satisfied were you with Robo?” (adapted from Bickmore et al., 2010). CA= Conversational agent. Standardized regression coefficients with 95% confidence intervals in parentheses. Higher satisfaction values indicate higher satisfaction with the CA interaction. Experience is experience with COPD in years since COPD diagnosis. Education is measured in years of formal education. CA Type is coded 0 = paternalistic, 1 = deliberative. Gender is coded 0 = male, 1 = female. Age in years. Experience in years since COPD diagnosis. Significant results are printed in bold font indicating that the 95% Confidence interval does not contain 0.

**Table S2.** This table describes the regression of CA type, demographics, and COPD related characteristics on participants' satisfaction (Item 2) with the CA interaction.

|  | Model 1 | Model 2 | Model 3 |
| --- | --- | --- | --- |
|  |  |  |  |
| Intercept | 0.039 | -0.070 | -0.044 |
|  | (-0.215, 0.292) | (-0.391, 0.251) | (-0.495, 0.406) |
| CA Type | -0.077 | 0.110 | 0.111 |
|  | (-0.436, 0.282) | (-0.366, 0.586) | (-0.514, 0.737) |
| Gender |  | 0.034 | 0.331 |
|  |  | (-0.556, 0.625) | (-0.495, 1.158) |
| Age |  | 0.169 | 0.322 |
|  |  | (-0.114, 0.453) | (-0.124, 0.769) |
| Education |  | 0.056 | 0.090 |
|  |  | (-0.219, 0.331) | (-0.289, 0.468) |
| Gender*CA Type |  | -0.284 | -**0.941** |
|  |  | (-1.086, 0.518) | (-1.990, 0.108) |
| Age*CA Type |  | -0.239 | **-0.585** |
|  |  | (-0.617, 0.138) | (-1.130, -0.041) |
| Education*CA Type |  | -0.111 | -0.075 |
|  |  | (-0.495, 0.273) | (-0.555, 0.405) |
| GOLD |  |  | 0.107 |
|  |  |  | (-0.249, 0.463) |
| COPD Literacy |  |  | 0.326 |
|  |  |  | (-0.153, 0.806) |
| Experience |  |  | -0.267 |
|  |  |  | (-0.645, 0.111) |
| GOLD*CA Type |  |  | 0.071 |
|  |  |  | (-0.428, 0.570) |
| COPD Literacy*CA Type |  |  | 0.189 |
|  |  |  | (-0.382, 0.759) |
| Experience* CA Type |  |  | 0.306 |
|  |  |  | (-0.213, 0.825) |
|  |  |  |  |
| Observations | 120 | 113 | 67 |
| R^2^ | 0.001 | 0.025 | 0.288 |

*Note.* Wording Item 2: “All things considered, I am very satisfied with Robo.” (adapted from Wixom & Todd, 2005). CA= Conversational agent. Standardized regression coefficients with 95% confidence intervals in parentheses. Higher satisfaction values indicate higher satisfaction with the CA interaction. Experience is experience with COPD in years since COPD diagnosis. Education is measured in years of formal education. CA Type is coded 0 = paternalistic, 1 = deliberative. Gender is coded 0 = male, 1 = female. Age in years. Experience in years since COPD diagnosis. Significant results are printed in bold font indicating that the 95% Confidence interval does not contain 0.

**Table S3.** This table describes the regression of CA type, demographics, and COPD related characteristics on participants' willingness to recommend the CA to a friend with COPD.

|  | Model 1 | Model 2 | Model 3 |
| --- | --- | --- | --- |
|  |  |  |  |
| Intercept | 0.098 | 0.104 | 0.255 |
|  | (-0.155, 0.351) | (-0.214, 0.422) | (-0.183, 0.694) |
| CA Type | -0.196 | -0.049 | -0.133 |
|  | (-0.554, 0.162) | (-0.521, 0.423) | (-0.742, 0.476) |
| Gender |  | -0.062 | 0.091 |
|  |  | (-0.647, 0.523) | (-0.714, 0.895) |
| Age |  | -0.071 | 0.107 |
|  |  | (-0.352, 0.210) | (-0.328, 0.541) |
| Education |  | -0.136 | -0.083 |
|  |  | (-0.409, 0.136) | (-0.451, 0.286) |
| Gender*CA Type |  | -0.386 | -0.646 |
|  |  | (-1.181, 0.409) | (-1.667, 0.376) |
| Age*CA Type |  | -0.094 | -0.463 |
|  |  | (-0.468, 0.280) | (-0.993, 0.067) |
| Education*CA Type |  | 0.068 | 0.080 |
|  |  | (-0.312, 0.448) | (-0.387, 0.547) |
| GOLD |  |  | 0.296 |
|  |  |  | (-0.051, 0.643) |
| COPD Literacy |  |  | 0.036 |
|  |  |  | (-0.431, 0.503) |
| Experience |  |  | -0.142 |
|  |  |  | (-0.510, 0.225) |
| GOLD*CA Type |  |  | -0.327 |
|  |  |  | (-0.813, 0.159) |
| COPD Literacy*CA Type |  |  | 0.451 |
|  |  |  | (-0.105, 1.006) |
| Experience* CA Type |  |  | 0.073 |
|  |  |  | (-0.432, 0.578) |
|  |  |  |  |
| Observations | 120 | 113 | 67 |
| R^2^ | 0.010 | 0.057 | 0.308 |

*Note.* CA= Conversational agent. Standardized regression coefficients with 95% confidence intervals in parentheses. Experience is experience with COPD in years since COPD diagnosis. Education is measured in years of formal education. CA Type is coded 0 = paternalistic, 1 = deliberative. Gender is coded 0 = male, 1 = female. Age in years. Experience in years since COPD diagnosis. Significant results are printed in bold font indicating that the 95% Confidence interval does not contain 0.

**Table S4.** This table describes the regression of CA type, demographics, and COPD related characteristics on participants' perceived relationship quality with the CA (Item 2).

|  | Model 1 | Model 2 | Model 3 |
| --- | --- | --- | --- |
|  |  |  |  |
| Intercept | -0.163 | -0.221 | -0.415 |
|  | (-0.414, 0.088) | (-0.539, 0.097) | (-0.919, 0.090) |
| CA Type | 0.326 | 0.340 | 0.535 |
|  | (-0.028, 0.681) | (-0.132, 0.813) | (-0.165, 1.235) |
| Gender |  | -0.034 | 0.463 |
|  |  | (-0.619, 0.552) | (-0.463, 1.388) |
| Age |  | 0.161 | 0.172 |
|  |  | (-0.120, 0.443) | (-0.328, 0.672) |
| Education |  | -0.130 | 0.068 |
|  |  | (-0.403, 0.142) | (-0.356, 0.492) |
| Gender*CA Type |  | 0.126 | -0.712 |
|  |  | (-0.670, 0.921) | (-1.887, 0.462) |
| Age*CA Type |  | -0.112 | -0.275 |
|  |  | (-0.486, 0.262) | (-0.884, 0.335) |
| Education*CA Type |  | 0.145 | 0.020 |
|  |  | (-0.236, 0.525) | (-0.518, 0.557) |
| GOLD |  |  | 0.226 |
|  |  |  | (-0.172, 0.625) |
| COPD Literacy |  |  | 0.378 |
|  |  |  | (-0.159, 0.915) |
| Experience |  |  | -0.005 |
|  |  |  | (-0.428, 0.418) |
| GOLD*CA Type |  |  | -0.135 |
|  |  |  | (-0.693, 0.424) |
| COPD Literacy*CA Type |  |  | -0.216 |
|  |  |  | (-0.855, 0.423) |
| Experience* CA Type |  |  | 0.191 |
|  |  |  | (-0.390, 0.772) |
| Observations | 120 | 113 | 67 |
| R^2^ | 0.027 | 0.058 | 0.148 |

*Note.* Wording Item 2: “I think, Robo liked me.” (adapted from Kiluk et al., 2014) CA= Conversational agent. Standardized regression coefficients with 95% confidence intervals in parentheses. Experience is experience with COPD in years since COPD diagnosis. Education is measured in years of formal education. CA Type is coded 0 = paternalistic, 1 = deliberative. Gender is coded 0 = male, 1 = female. Age in years. Experience in years since COPD diagnosis. Significant results are printed in bold font indicating that the 95% Confidence interval does not contain 0.
